# Association between acupuncture treatment exposure and mortality in patients with heart failure: a nationwide cohort study

**DOI:** 10.3389/fcvm.2025.1461302

**Published:** 2025-07-15

**Authors:** Hyungsun Jun, Hanbit Jin, Haerim Kim, Jungtae Leem

**Affiliations:** ^1^ Department of Diagnostics, College of Korean Medicine, Wonkwang University, Iksan, Republic of Korea; ^2^ Department of Preventive Medicine, College of Korean Medicine, Dongshin University, Naju, Republic of Korea; ^3^ Department of Statistics, University of Seoul, Seoul, Republic of Korea; ^4^ Research Center of Traditional Korean Medicine, College of Korean Medicine, Wonkwang University, Iksan, Republic of Korea; ^5^ Department of Il-won Integrated Medicine, Wonkwang University Korean Medicine Hospital, Iksan, Republic of Korea

**Keywords:** acupuncture, traditional Korean medicine, cohort study, heart failure, national health insurance

## Abstract

**Introduction:**

Patients with heart failure (HF) require continuous management, creating a need for alternative treatments to reduce mortality. In Korea, acupuncture treatment is covered by national health insurance, offering accessible care without financial burden. Hence, this study utilized claims data from the Korean National Health Insurance Service to examine the association between acupuncture exposure and mortality in patients with HF.

**Methods:**

Adults aged 20 years or older with newly diagnosed HF were included. Patients who received two or more acupuncture sessions within one year of diagnosis comprised the acupuncture treatment-exposed group (AT group), while those who received none were classified as the non-exposed group (non-AT group). Propensity score matching was used to generate a balanced 1:1 matched cohort. From one year after diagnosis, the study tracked circulatory and all-cause mortality over a five-year period, performing survival and dose-response analyses.

**Results:**

Each group included 4,315 patients, and the AT group showed significantly lower adjusted hazard ratios (aHRs) for circulatory disease-related mortality (0.79; 95% CI: 0.67–0.94) and all-cause mortality (0.73; 95% CI: 0.66–0.81). A clear dose-response relationship was observed, with greater exposure to acupuncture associated with lower aHRs.

**Discussion:**

Acupuncture within one year of HF diagnosis correlated with lower mortality from circulatory diseases and all causes. Future studies should adopt prospective and methodologically rigorous designs to validate the findings of this study.

## 1 Introduction

Heart failure (HF) was defined by a committee in 2021 as “a clinical syndrome with symptoms and/or signs caused by a structural and/or functional cardiac abnormality and corroborated by elevated natriuretic peptide levels and/or objective evidence of pulmonary or systemic congestion” (1). Although the incidence of HF has exhibited stable or declining trends over time, advancements in treatment and increased life expectancy have led to an increase in its prevalence (2). In 2017, the age-standardized prevalence of HF was reported to be the highest in Central Europe, North Africa, and the Middle East, ranging from 1,133 to 1,196 cases per 100,000 population. Conversely, Eastern Europe and Southeast Asia have reported the lowest rates, ranging from 498 to 595 cases per 100,000 population (3). Examining the age-standardized prevalence in Korea between 2002 and 2018, a 116% increase in males (from 1,106 to 2,386 cases per 100,000) and a 70% increase in females (from 1,270 to 2,164 cases per 100,000) were noted (4). Regarding the mortality rate of HF, studies on the elderly and adults reported a 1-year mortality rate of 33% and 24%, respectively (5). An analysis of 39,982 patients hospitalized for HF in the United States between 2005 and 2009 reported a median survival of 2.1 years and a 5-year mortality rate of 75.4% (6). A systematic review investigating the causes of death in HF found that over 50% of deaths were attributed to cardiovascular diseases in 14 of 19 studies (7). As indicated by prior research, individuals with HF typically have a mortality rate exceeding 70% within 5 years of diagnosis, primarily due to cardiovascular diseases.

HF is categorized into three types based on the left ventricular ejection fraction: reduced, mildly reduced, and preserved (2). The treatment guidelines for HF vary depending on the patient's ejection fraction; however, guideline-directed medical therapy recommends pharmacological treatments such as renin-angiotensin system inhibitors, beta-blockers, mineralocorticoid receptor antagonists, and sodium-glucose cotransporter 2 inhibitors (8–10). While these medications generally provide more benefits than side effects, there are instances where patients cannot undergo treatment because of medication-related side effects such as low heart rate, hypotension, renal impairment, and hyperkalemia (11–15). In cases where pharmacological treatment is ineffective or the goal is to prevent acute cardiac arrest, surgical interventions such as implantable cardioverter-defibrillator or cardiac resynchronization therapy may be considered (4). Despite medical and surgical interventions, heart transplantation is ultimately considered when improvement is no longer expected. However, the scarcity of donors and strict contraindications to heart transplantation pose challenges in obtaining a transplant. Even for those who undergo heart transplantation, risk of complications such as malignant tumors, infections, and cardiac allograft vasculopathy exists (4, 16). Therefore, alternative therapies are needed to prevent death through the continuous management of patients with HF.

In East Asia, traditional medicine, including acupuncture and herbal medicine, is widely used to treat various cardiovascular conditions (17–19), and ongoing research has explored its application to HF (20–22). The American Heart Association, in analyzing the clinical efficacy of acupuncture in HF, reported that it is believed to affect neural impulses, release endorphins, opioids, or both, and result in sympathetic stimulation of neurotransmitters with no serious adverse effects observed (23). Two randomized controlled trials (RCTs) involving acupuncture in patients with HF revealed positive effects on 6-minute walk distance, improvements in quality of life, and increased heart rate variability. Additionally, acupuncture was reported to have a beneficial effect on alleviating the rapid increase in sympathetic nervous system activity during mental stress (24, 25). A literature review of HF and acupuncture has highlighted broad neurohumoral mechanisms, suggesting improvements in cardiovascular function and clinical indicators, along with sympatholytic, vasodilatory, and cardioprotective effects (26). Systematic reviews have indicated that acupuncture is associated with reduced readmission rates and shortened intensive care unit stay. Studies have also reported significant improvements in key indicators of cardiac function, such as left ventricular ejection fraction and cardiac output, following acupuncture and moxibustion therapy (27, 28). While diverse short-term clinical studies on acupuncture in HF exist, they face limitations in terms of generalizability, small sample sizes, and the need for long-term observations owing to the nature of cardiovascular diseases. Therefore, retrospective cohort studies utilizing accumulated large-scale data over an extended period are needed to overcome these limitations.

The National Health Insurance Service (NHIS) in South Korea provides researchers with access to comprehensive claims data, facilitating the advancement of healthcare research. Real-world data (RWD) derived from routine clinical practice enables large-scale, long-term follow-up of target populations. A study using Taiwan's NHIS data reported improved survival in patients with HF who concurrently received traditional Chinese medicine (TCM) (29). However, in Taiwan, a major limitation is that inpatient TCM treatments are not covered by insurance (30). A scoping review of observational studies using RWD found that East Asian traditional medicine was associated with reduced mortality and readmission in HF patients, although 11 of the 12 studies focused on herbal interventions, with none specifically addressing acupuncture (31). In addition, a systematic review of RCTs on acupuncture in HF showed symptomatic improvement but no significant impact on mortality (27, 28). To address these gaps, we conducted a nationwide retrospective cohort study to evaluate the impact of acupuncture treatment exposure on mortality outcomes in patients with HF. Survival analysis was performed using national claims data from the Korean NHIS.

## 2 Methods

**Figure 1:**
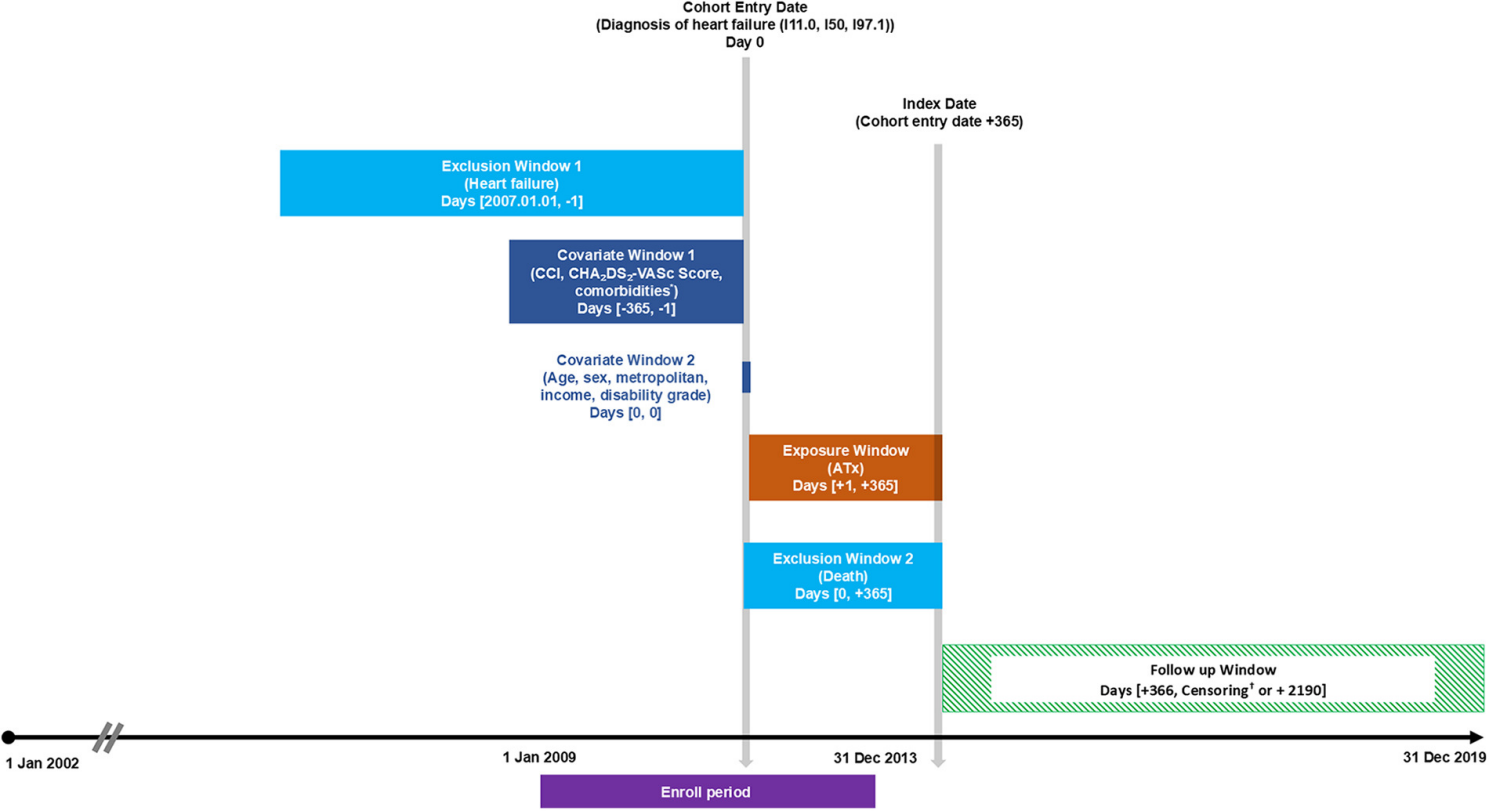
Exposure-based cohort entry restricted to adherent users. ATx, acupuncture treatment; CCI, Charlson comorbidity index. *Comorbidities included hypertension, diabetes mellitus, dyslipidemia, myocardial infarction, peripheral artery disease, chronic obstructive pulmonary disease, and cancer. ^†^Censoring occurred at the first event—either death or the conclusion of the study period. Adapted with permission from “Exposure-based Cohort Entry Restricted to Adherent Users” by Schneeweiss S et al., licensed under CC BY 4.0.

For the retrospective cohort analysis of adherent users, the study visualized exposure, covariates, exclusion criteria, and follow-up periods for each timeframe (Figure 1) (32).

### 2.1 Date sources

As of March 2023, the NHIS in South Korea has a membership of 51,404,000 people, covering over 99% of the population. The NHIS provides services in the form of goods or cash for the prevention, diagnosis, treatment, rehabilitation, childbirth, death, and health promotion of diseases and injuries of its subscribers and their dependents in accordance with regulations (33). The sample cohort database provided by the NHIS was used for this study, comprising a sample of 1 million individuals, approximately 2% of the entire population, who have maintained health insurance or medical aid eligibility annually since 2006. Stratified sampling was conducted based on sex, age, income level, and region to ensure representativeness through systematic stratified random sampling with proportional allocation within each stratum. Data from 2002 to 2019 were made available for the analysis (34). The data included sociodemographic characteristics, medical history, health behaviors, physical measurements, test results, medical utilization, death, healthcare facilities, and long-term care service information. This study was approved by the Institutional Review Board at Wonkwang University (WKIRB-202310-SB-075).

### 2.2 Study participants

The study participants were selected based on the following criteria. First, individuals aged ≥20 years who received a new diagnosis of HF between January 1, 2009, and December 31, 2013, within the enrollment period were included. Second, following previous research, the diagnosis of HF was defined using disease codes from the 10th revision of the International Classification of Diseases (ICD-10), specifically I11.0 [hypertensive heart disease with (congestive) heart failure], I50 (heart failure), and I97.1 (other functional disturbances following cardiac surgery). The primary or secondary diagnosis had to include these disease codes, and for diagnostic accuracy, patients were considered to have HF if they had visited outpatient clinics two or more times or were hospitalized at least once within one year of the cohort entry date. The exclusion criteria were as follows. To eliminate patients already diagnosed with HF at the time of registration, a washout period was established from January 1, 2007, to December 31, 2008. Patients who had only one outpatient visit for HF treatment within one year of the cohort entry date were also excluded. Additionally, individuals who died within one year of the diagnosis date or had missing demographic information were not included in the study.

### 2.3 Exposure intervention

Using the 1-year landmark methodology, patients newly diagnosed with HF during the enrollment period were assigned to the acupuncture treatment-exposed group (AT group) if they underwent acupuncture two or more times within 1 year from diagnosis. Those who did not receive acupuncture within 1 year of diagnosis were categorized into the non-exposed acupuncture treatment group (non-AT group). Patients who received acupuncture treatment only once did not meet the criteria for either the AT group or the non-AT group and were therefore excluded. As there are no clear criteria for the number of acupuncture sessions and an appropriate landmark period for exposure assessment, the research team decided based on internal consensus and referred to existing studies (35–37). Acupuncture treatment was verified using the codes 40011 (single body part), 40012 (multiple body parts), 40030 (intra-orbital cavity), 40040 (intranasal sinus), 40050 (intra-peritoneal cavity), 40060 (intra-articular), 40070 (interspace of spinous process), 40080 (penetration), and 40091 (electro-acupuncture). Patients who received acupuncture, regardless of the acupoint or diagnostic relevance, were assigned to the AT group.

### 2.4 Outcomes

The index date was defined as one year after the initial diagnosis of HF, marking the beginning of the follow-up period. Participants were followed for up to five years or until the occurrence of clinical endpoints, whichever came first. Clinical endpoints were defined as death from any cause or death due to circulatory system diseases, identified using ICD-10 codes I00–I99. Mortality data were confirmed by cross-referencing population death registration records from Statistics Korea with individual identification numbers.

### 2.5 Potential confounders

Utilizing propensity score (PS) matching enables the creation of well-matched groups while minimizing the impact of confounding factors. In this study, the statistical method of PS matching was employed to match individuals between the two groups at a 1:1 ratio. Multivariable logistic regression was used to estimate the PS model, where each participant's estimated probability of being in the group, based on the given covariates, corresponded to the PS. Basic characteristics, including age (<65, 65–74, ≥75 years), sex (male, female), residential area (metropolitan, urban, rural), income (bottom 0–3, middle 4–7, top 8–10), and disability grade (non-disabled, mild, severe), were considered as covariates based on the cohort entry date. One year before the cohort entry date, the CHA_2_DS_2_-VAS_C_ score (0, 1, 2, ≥3) was employed as a predictor for vascular dysfunction and cardiovascular events (38, 39), and the Charlson comorbidity index (CCI, 0, 1, 2, ≥3) was utilized to gauge underlying conditions (40, 41). Additionally, various comorbidities, including hypertension, diabetes mellitus, dyslipidemia, myocardial infarction, peripheral artery disease, chronic obstructive pulmonary disease, and cancer, were taken into consideration (Supplementary Table S1). The criterion for acceptability was set with a standardized mean difference (SMD) value of ≤0.05. A caliper of 0.01 was used, and matching was performed using the greedy nearest-neighbor method.

### 2.6 Statistical analysis

Descriptive statistics were used to characterize the baseline characteristics. Continuous variables are presented as mean ± standard deviation (SD) and categorical variables as absolute numbers and percentages (%). The t-test was used to compare continuous variables between the two groups based on normality, whereas the chi-square and Fisher's exact tests were used to assess categorical variables. Kaplan–Meier curves were used to visualize the clinical outcomes, and the proportional hazards assumption was tested based on Schoenfeld residuals. Given that the proportional hazards assumption was met, the Cox proportional hazards model was employed for analysis. The incidence rates (IR) for all-cause mortality and mortality due to circulatory system diseases are presented by providing the total number of events and total person-years. The number of events per 100 person-years is reported as IR/100 person-years. Hazard ratios (HRs) and 95% confidence intervals (CIs) were estimated using the Cox proportional hazards model. The results are presented both before and after adjusting for age, sex, residential area, income, disability grade, CHA_2_DS_2_-VAS_C_ score, CCI, and comorbidities (hypertension, diabetes mellitus, dyslipidemia, myocardial infarction, peripheral artery disease, chronic obstructive pulmonary disease, and cancer).

For the dose-response analysis, the number of acupuncture sessions was categorized into quartiles for survival analysis. To mitigate the arbitrariness of categorization, the same variable was additionally analyzed as a continuous variable. The restricted cubic spline (RCS) method was applied using the R packages rms and changepoint to explore trends in HRs for mortality associated with increasing acupuncture frequency (42, 43). Subgroup analyses were conducted based on covariates including age, sex, residential area, income level, CHA_2_DS_2_-VASc score, and CCI, with results presented in a forest plot. For the sensitivity analysis, only patients in the non-AT group who had not received any acupuncture treatment even after one year from the HF diagnosis were included, and survival analysis was conducted to assess the robustness of the findings. A two-sided *p*-value of <0.05 was considered statistically significant. Statistical analyses and visualizations were conducted using R version 4.2.1 (The R Foundation, https://www.R-project.org) and SAS 9.4 version (SAS Institute Inc., Cary, NC, USA).

## 3 Results

### 3.1 Patient characteristics

**Figure 2:**
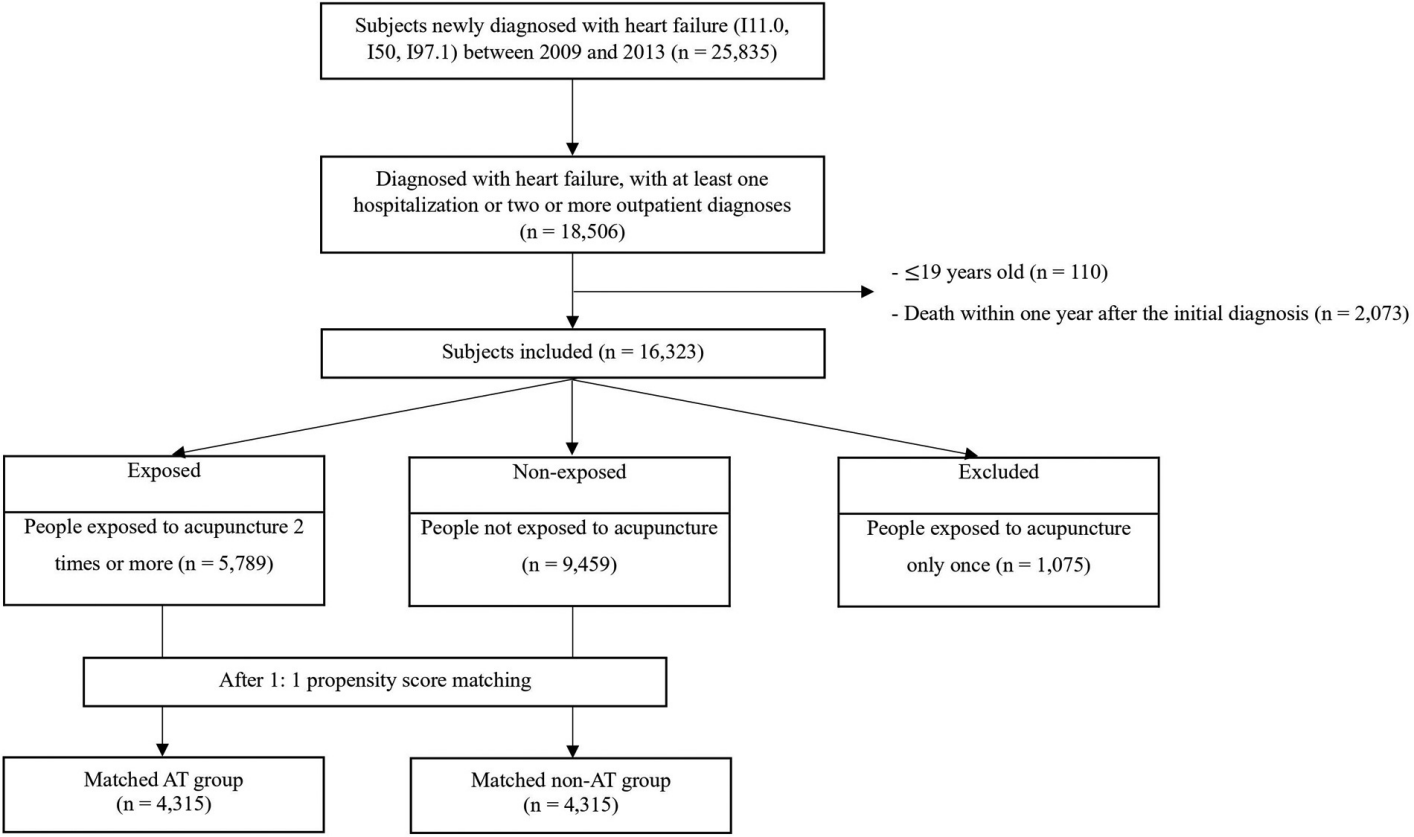
Flowchart of sample selection. AT, acupuncture treatment-exposed group; non-AT, non-exposed acupuncture treatment group.

Of 25,835 patients newly diagnosed with HF, 16,323 were selected based on the exclusion criteria. After evaluating the exposure, 5,789 were classified to the AT group, and 9,459 into the non-AT group. Through 1:1 PS matching, each group comprised 4,315 patients (Figure 2). Before PS matching, the mean age was higher in the AT group than in the non-AT group (65.19 vs. 67.54 years), and the sex ratio was 49.7:50.3 in the non-AT group and 34.6:65.4 in the AT group, indicating a higher proportion of females in the AT group. In terms of residential area, rural residents accounted for the largest percentage in the AT group (44.4%). CCI (mean 2.46 vs. 2.80) and CHA_2_DS_2_-VAS_C_ score (mean 2.89 vs. 3.32) indicated higher scores in the AT group, suggesting a greater burden of underlying conditions. The prevalence of comorbidities, such as hypertension, dyslipidemia, and peripheral artery disease, was higher in the AT group than in the non-AT group. After PS matching, the covariates in both groups were evenly distributed with SMDs below 0.05 (Table 1).

**Table 1:** Patient demographics and clinical characteristics.

Characteristics	Before PS matching			After PS matching		
	Non-AT group (*n* = 9,459)	AT group (*n* = 5,789)	SMD	Non-AT group (*n* = 4,315)	AT group (*n* = 4,315)	SMD
Age, mean (SD)	65.19 (13.85)	67.54 (11.86)	0.182	66.31 (13.64)	66.74 (12.08)	0.033
Age, *n* (%)			0.198			0.051
<65	4,208 (44.5)	2,035 (35.2)		1,707 (39.6)	1,663 (38.5)	
65–74	2,589 (27.4)	1,974 (34.1)		1,305 (30.2)	1,406 (32.6)	
≥75	2,662 (28.1)	1,780 (30.7)		1,303 (30.2)	1,246 (28.9)	
Sex, *n* (%)			0.308			0.002
Male	4,700 (49.7)	2,005 (34.6)		1,656 (38.4)	1,652 (38.3)	
Female	4,759 (50.3)	3,784 (65.4)		2,659 (61.6)	2,663 (61.7)	
Residential area, *n* (%)		0.114			0.052	
Metropolitan	3,844 (40.7)	2,032 (35.1)		1,704 (39.5)	1,630 (37.8)	
Urban	1,771 (18.7)	1,185 (20.5)		786 (18.2)	870 (20.2)	
Rural	3,838 (40.6)	2,567 (44.4)		1,825 (42.3)	1,815 (42.1)	
Income, *n* (%)			0.066			0.018
Medical aid & Low	1,877 (22.3)	1,110 (21.7)		915 (21.2)	935 (21.7)	
Middle	2,041 (24.3)	1,121 (21.9)		977 (22.6)	948 (22.0)	
High	4,490 (53.4)	2,891 (56.4)		2,423 (56.2)	2,432 (56.4)	
Disability grade, *n* (%)						0.012
Non-disabled	7,786 (82.3)	4,814 (83.2)		3,728 (86.4)	3,719 (86.2)	
Mild	1,004 (10.6)	648 (11.2)		394 (9.1)	408 (9.5)	
Severe	669 (7.1)	327 (5.6)		193 (4.5)	188 (4.4)	
CHA_2_DS_2_-VAS_C_ score, mean (SD)	2.89 (1.67)	3.32 (1.66)	0.257	3.15 (1.64)	3.14 (1.64)	0.004
CHA_2_DS_2_-VAS_C_ score, *n* (%)			0.246			0.026
0	565 (6.0)	177 (3.1)		159 (3.7)	154 (3.6)	
1	1,559 (16.5)	671 (11.6)		574 (13.3)	589 (13.7)	
2	2,007 (21.2)	1,051 (18.2)		822 (19.0)	859 (19.9)	
≥3	5,328 (56.3)	3,890 (67.2)		2,760 (64.0)	2,713 (62.9)	
CCI, mean (SD)	2.46 (2.25)	2.80 (2.19)	0.154	2.57 (2.17)	2.62 (2.17)	0.020
CCI, *n* (%)			0.209			0.016
0	1,772 (18.7)	696 (12.0)		640 (14.8)	620 (14.4)	
1	2,101 (22.2)	1,196 (20.7)		948 (22.0)	970 (22.5)	
2	1,741 (18.4)	1,116 (19.3)		854 (19.8)	850 (19.7)	
≥3	3,845 (40.6)	2,781 (48.0)		1,873 (43.4)	1,875 (43.5)	
Comorbidities, *n* (%)						
Hypertension	6,695 (70.8)	4,292 (74.1)	0.075	3,160 (73.2)	3,180 (73.7)	0.010
Diabetes mellitus	3,023 (32.0)	1,901 (32.8)	0.019	1,398 (32.4)	1,368 (31.7)	0.015
Dyslipidemia	3,818 (40.4)	2,600 (44.9)	0.092	1,854 (43.0)	1,794 (41.6)	0.028
MI	583 (6.2)	308 (5.3)	0.036	215 (5.0)	222 (5.1)	0.007
PAD	1,406 (14.9)	1,170 (20.2)	0.141	726 (16.8)	719 (16.7)	0.004
COPD	561 (5.9)	364 (6.3)	0.015	217 (5.0)	223 (5.2)	0.006
Cancer	612 (6.5)	357 (6.2)	0.012	243 (5.6)	263 (6.1)	0.020

AT group, patients with acupuncture exposure ≥2 times within the first year post-diagnosis; CCI, Charlson comorbidity index; COPD, chronic obstructive pulmonary disease; MI, myocardial infarction; Non-AT group, subjects not exposed to acupuncture within the first year after diagnosis; PAD, peripheral artery disease; PS, propensity score; SD, standard deviation; SMD, standardized mean difference.

### 3.2 Rates of circulatory system disease and all-cause mortality

**Figure 3:**
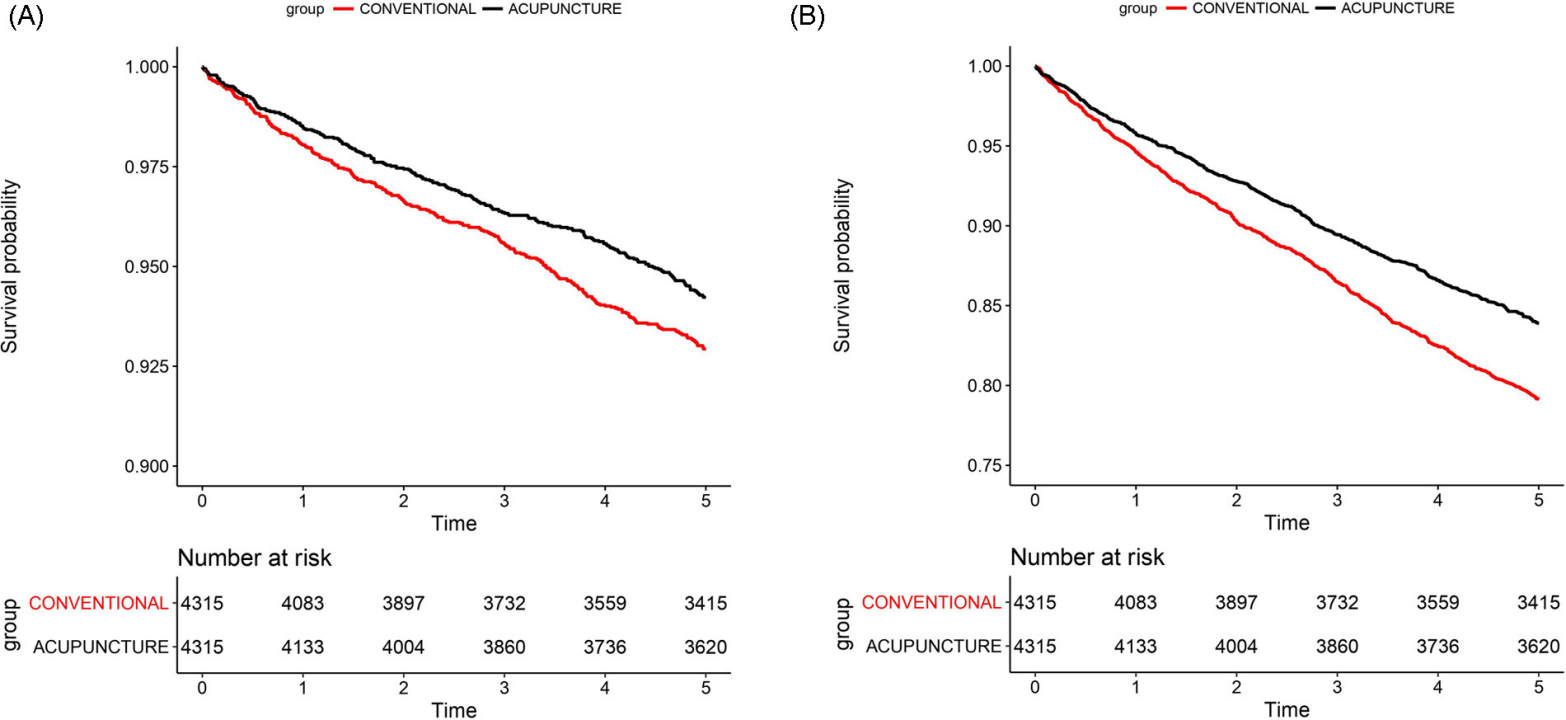
Kaplan–meier curves. Kaplan–Meier curves showing the survival probability in the acupuncture and non-acupuncture treatment groups after PS matching. Both A and B show *p*-values <0.05, indicating a significant difference. **(A)** Circulatory system disease-related mortality and **(B)** all-cause mortality.

After PS matching, the mortality rates due to circulatory system diseases were 1.19/100 person-years in the AT group and 1.48/100 person-years in the non-AT group. The all-cause mortality rate was 3.53/100 person-years in the AT group and 4.7/100 person-years in the non-AT group. Therefore, the incidence of mortality due to circulatory system diseases and all-causes was lower in the AT group than that in the non-AT group. The AT group showed significantly lower adjusted HR (aHR) for mortality due to circulatory system diseases (aHR, 0.79, 95% CI, 0.67–0.94) and all-cause mortality (aHR, 0.73, 95% CI, 0.66–0.81) compared to the non-AT group (Table 2). Kaplan–Meier curves were used to depict survival probabilities for circulatory system disease-related and all-cause mortality after PS matching (Figure 3).

**Table 2:** Hazard ratios for mortality in heart failure patients.

Mortality	Event, *n* (%)	IR (per 100PY)	PS-matched HR (95% CI)	*P*	Adjusted HR (95% CI)^a^	*P*
Circulatory system disease						
Non-AT group	283 (6.56)	1.48	1		1	
AT group	235 (5.45)	1.19	0.81 (0.68–0.96)	0.02	0.79 (0.67–0.94)	0.01
All-cause						
Non-AT group	900 (20.86)	4.7	1		1	
AT group	695 (16.11)	3.53	0.75 (0.68–0.83)	<0.001	0.73 (0.66–0.81)	<0.001

^a^ Cox proportional hazards models were used to estimate hazard ratios, adjusted for age, sex, residential area, income level, disability grade, CHA_2_DS_2_-VASc score, Charlson Comorbidity Index, and comorbidities (hypertension, diabetes mellitus, dyslipidemia, myocardial infarction, peripheral artery disease, chronic obstructive pulmonary disease, and cancer).

AT group, patients with acupuncture exposure ≥2 times within the first year post-diagnosis; CI, confidence interval; HR, hazard ratios; IR, incidence rate; Non-AT group, subjects not exposed to acupuncture within the first year post-diagnosis; PS, propensity score; PY, person-year.

### 3.3 Dose-response analysis

**Figure 4:**
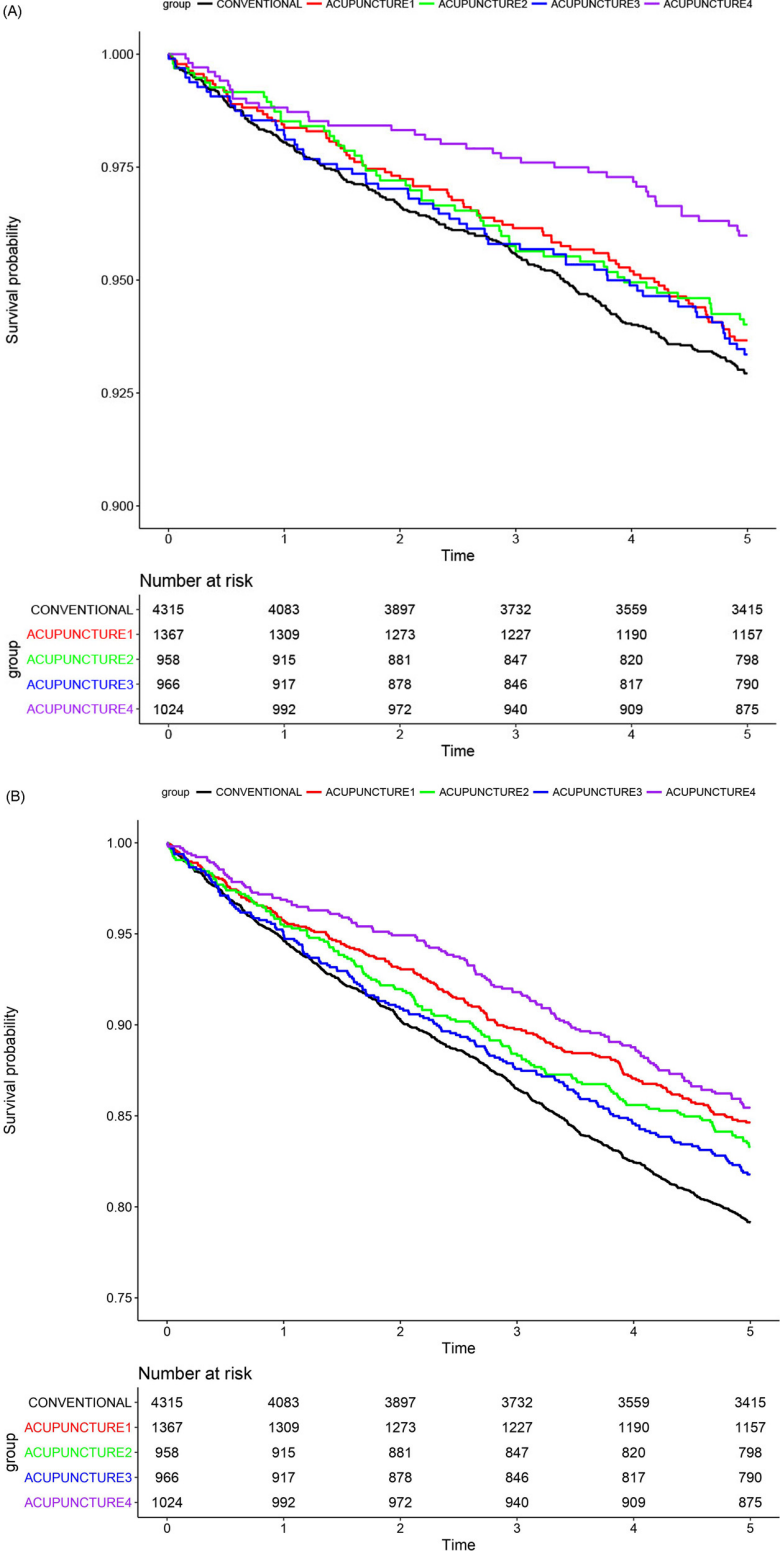
Kaplan–meier curves of dose-response analysis. ACUPUNCTURE1, acupuncture exposure <4 times; ACUPUNCTURE2, acupuncture exposure ≥4 times and <8 times; ACUPUNCTURE3, acupuncture exposure ≥8 times and <17 times; ACUPUNCTURE4, acupuncture exposure ≥17 times. Kaplan–Meier curves showing the survival probability in the four groups, stratified by the number of acupuncture treatments, along with the non-acupuncture treatment group after PS matching. Both A and B show *p*-values <0.05, indicating a significant difference. **(A)** Circulatory system disease-related mortality and **(B)** all-cause mortality.

To conduct a dose-response analysis, the number of acupuncture treatment sessions within 1 year after onset was categorized into quartiles: Q1 (4 sessions), Q2 (8 sessions), and Q3 (17 sessions). The AT group was then divided into four subgroups (AT-1, fewer than four sessions; AT-2, four or more but fewer than eight sessions; AT-3, eight or more but fewer than 17 sessions; and AT-4, 17 or more sessions). These subgroups were compared with the non-AT group to examine the differences in mortality rates. Mortality due to circulatory system diseases was significantly lower only in the AT-4 group, with an aHR of 0.47 (95% CI, 0.34–0.66). However, a decreasing trend in aHR was observed with an increase in the number of acupuncture sessions. For all-cause mortality, aHRs were significantly lower in all four AT groups (AT-1: aHR, 0.78, 95% CI, 0.67–0.91; AT-2: aHR, 0.80, 95% CI, 0.68–0.95; AT-3: aHR, 0.79, 95% CI, 0.67–0.93; AT-4: aHR, 0.57, 95% CI, 0.48–0.67) (Table 3, Figure 4). Analyzing the number of acupuncture sessions in the AT group as a continuous variable revealed a continuous decreasing trend in mortality with an increasing number of acupuncture sessions. The aHR for circulatory system diseases in patients with 30 or more sessions was 0.47 (95% CI, 0.31–0.73), and similarly, the aHR for all-cause mortality in those with 30 or more sessions was 0.58 (95% CI, 0.47–0.72) (Supplementary Tables S2, S3). Applying the RCS curve method, the mortality due to circulatory system diseases exhibited a decreasing aHR for up to 30 sessions, followed by a slight increase. After approximately 70 sessions, the 95% CI included 1, indicating no significant difference. The all-cause mortality rate displayed a J-shaped curve, with a decreasing aHR up to 30 sessions and a gradual increase afterward. No statistically significant differences were observed after 117 sessions (Supplementary Figure S1).

**Table 3:** Hazard ratios for mortality stratified by quartiles of acupuncture treatment exposure.

Mortality	Total, *N*	Event, *n* (%)	IR (per 100PY)	PS-matched HR (95% CI)	*P*	Adjusted HR (95% CI)^a^	*P*
Circulatory system disease							
Non-AT group	4,315	283 (6.56)	1.48	1		1	
AT group							
AT-1	1,367	82 (6.00)	1.31	0.89 (0.69–1.13)	0.34	1.00 (0.78–1.28)	0.99
AT-2	958	54 (5.64)	1.24	0.84 (0.63–1.13)	0.24	0.87 (0.65–1.18)	0.38
AT-3	966	60 (6.21)	1.38	0.94 (0.71–1.24)	0.64	0.86 (0.65–1.14)	0.29
AT-4	1,024	39 (3.81)	0.82	0.56 (0.40–0.78)	<0.001	0.47 (0.34–0.66)	<0.001
All-cause							
Non-AT group	4,315	900 (20.86)	4.7	1		1	
AT group							
AT-1	1,367	210 (15.36)	3.35	0.71 (0.61–0.83)	<0.001	0.78 (0.67–0.91)	<0.001
AT-2	958	160 (16.70)	3.68	0.78 (0.66–0.93)	0.01	0.80 (0.68–0.95)	0.01
AT-3	966	176 (18.22)	4.06	0.86 (0.73–1.02)	0.07	0.79 (0.67–0.93)	0.01
AT-4	1,024	149 (14.55)	3.13	0.67 (0.56–0.79)	<0.001	0.57 (0.48–0.67)	<0.001

^a^ Cox proportional hazards models were used to estimate hazard ratios, adjusted for age, sex, residential area, income level, disability grade, CHA_2_DS_2_-VASc score, Charlson Comorbidity Index, and comorbidities (hypertension, diabetes mellitus, dyslipidemia, myocardial infarction, peripheral artery disease, chronic obstructive pulmonary disease, and cancer).

AT group, patients with acupuncture exposure ≥2 times within the first year post-diagnosis; AT-1, acupuncture exposure <4 times; AT-2, acupuncture exposure ≥4 times and <8 times; AT-3, acupuncture exposure ≥8 times and <17 times; AT-4, acupuncture exposure ≥17 times; CI, confidence interval; HR, hazard ratios; IR, incidence rate; Non-AT group, subjects not exposed to acupuncture within the first year post-diagnosis; PS, propensity score; PY, person-year.

### 3.4 Subgroup analysis

Subgroup analysis revealed that the association between acupuncture and reduced mortality varied across patient groups. For circulatory system disease-related mortality, lower aHRs were observed among patients aged ≥75 years, females, individuals with a CCI ≥3 or CHA_2_DS_2_-VASc score ≥3, those in higher income tiers, urban or metropolitan residents, and non-disabled individuals. For all-cause mortality, relatively lower aHRs were also observed among individuals aged ≥65 years, females, individuals with higher income, and those with no or mild disability. However, formal tests for interaction were not performed, and some subgroups included a limited number of events. Therefore, these findings should be interpreted as exploratory trends rather than definitive evidence of effect modification across subgroups (Supplementary Figure S2).

### 3.5 Sensitivity analysis

For sensitivity analysis, following PS matching, each group comprised 1,857 individuals (Supplementary Figure S3, Table S4). The AT group exhibited a significantly lower aHR for mortality due to circulatory system diseases than the non-AT group, with an aHR of 0.47 (95% CI, 0.37–0.60). Similarly, the aHR for all-cause mortality was notably lower at 0.43 (95% CI, 0.37–0.49) (Supplementary Figure S4, Table S5). The dose-response analysis revealed a consistent trend, where increasing exposure frequency corresponded to decreasing aHR (Supplementary Figure S5, S6, Tables S6–S8). Subgroup analysis indicated that patients with a severe disability grade or a CHA_2_DS_2_-VASc score of 2 showed HRs >1, although these results were not statistically significant. All other subgroups demonstrated HRs < 1, though statistical significance was not achieved in some cases (Supplementary Figure S7).

## 4 Discussion

### 4.1 Summary of findings

We conducted a retrospective cohort analysis using the NHIS sample cohort database to explore the association between acupuncture exposure and mortality in patients with HF. Patients newly diagnosed with HF between 2009 and 2013 were divided into those who were exposed and those who were not exposed to acupuncture treatment within one year of diagnosis. Through PS matching, resulting in 4,315 individuals in each group, the AT group exhibited significantly lower mortality hazards for circulatory system diseases and all-cause mortality over a 5-year follow-up period. Dose-response analysis, considering the acupuncture exposure frequency as both categorical and continuous, showed that only the highest exposure group (AT-4, with 17 or more sessions) had a significantly lower mortality hazards for circulatory system diseases than the non-AT group. However, all-cause mortality was significantly lower in all exposure groups. Analysis of continuous variables also indicated a decreasing trend in mortality hazards with increasing exposure frequency. Using the RCS curve method to identify cutoff values for the number of acupuncture sessions, both circulatory system and all-cause mortality consistently decreased for up to 30 sessions, followed by a slight increase. Subgroup analysis revealed that in some groups, the HR was >1; however, these differences were not statistically significant.

Korea benefits from a dual healthcare system in which traditional Korean medicine is covered by insurance, offering patients accessibility without substantial financial strain (44). An analysis of annual traditional Korean medicine utilization revealed higher usage among females, older individuals, rural residents, and those with lower incomes (45). Before PS matching, significant differences were observed in age, sex, residence, income, disability severity, CHA_2_DS_2_-VAS_C_ scores, and CCI between the AT and non-AT groups. Specifically, by confirming that the AT group had a higher proportion of individuals aged 65 years or older, females, rural residents, and higher-income patients, it was observed that except for income level, the previously reported frequency of use groups remained consistent. Elevated CHA_2_DS_2_-VAS_C_ and CCI scores suggest an increased risk of vascular dysfunction and cardiovascular disease, reflecting a higher overall burden of comorbid conditions. Higher scores were proportionally more prevalent in the AT group than in the non-AT group, suggesting a higher likelihood of acupuncture treatment for individuals with more severe conditions. To address intergroup differences, PS matching comprised 4,315 individuals in each group. However, during sensitivity analysis, the matched sample size decreased to 1,857 individuals, suggesting potential distinctions between those receiving continuous acupuncture treatment and those who are not.

The aHRs for mortality due to circulatory system diseases and all-cause mortality were significantly lower in the AT group, with values of 0.79 and 0.73, respectively. Additionally, a decreasing trend in aHR was observed with an increasing number of acupuncture treatment sessions, reaching 0.47 and 0.58 for mortality due to circulatory system diseases and all-cause mortality, respectively, with 30 or more sessions. In the sensitivity analysis in which patients who never received acupuncture treatment after HF diagnosis were set as the control group, lower aHRs were observed for mortality due to circulatory system diseases (0.47) and all-cause mortality (0.43). Furthermore, when adjusting for acupuncture exposure of 30 or more sessions in the AT group, the aHRs for mortality decreased to 0.27 for circulatory system diseases and 0.36 for all-cause mortality. In the sensitivity analysis, the mortality rate in the non-AT group was approximately 1.5 times higher than that observed in the original analysis. This could be interpreted as, in the original analysis, the non-AT group was defined only based on not being exposed to acupuncture within one year after diagnosis. Consequently, it is presumed that patients exposed later were also included, leading to a lower mortality rate compared to the sensitivity analysis. However, another plausible explanation is that including patients with extremely poor health who could not receive acupuncture treatment in the sensitivity analysis could result in worse outcomes. Further research is required to validate this hypothesis. Nevertheless, sensitivity analysis provided additional evidence supporting the association between acupuncture treatment in patients with HF and decreased mortality hazards. However, as this is an observational study, it cannot be concluded whether the mortality reduction in the AT group was directly due to acupuncture exposure or influenced by other factors. Efforts were made to minimize the impact of comorbidities on mortality through matching and hazard adjustment, but other risk factors, such as lifestyle habits, were not accounted for, underscoring the need for further research to address these limitations.

### 4.2 Recommendations for subsequent studies and implications for clinical practice

In the subgroup analysis, consistent reductions in mortality were not observed across all patient groups. However, relatively lower aHRs were noted among patients aged ≥75 years, females, individuals with higher comorbidity burden, higher income levels, those residing in urban or metropolitan areas, and those without or with only mild disability. While these patterns may suggest a potential differential response to acupuncture in certain HF subpopulations, formal statistical tests for interaction were not conducted, and the findings should therefore be considered descriptive and hypothesis-generating. Previous studies have also explored variation in acupuncture effectiveness by patient characteristics. For instance, clinical trials involving patients with chronic pain have reported greater effects among females, although meta-analyses have shown inconsistent modification of treatment effects by sex (46, 47). In contrast, a retrospective cohort study investigating acupuncture for secondary prevention of myocardial infarction in stroke patients found no significant differences by age or sex (48). These mixed findings suggest that treatment response may vary depending on the clinical context and population. To date, limited research has examined heterogeneous treatment effects of acupuncture in patients with HF. Although our findings suggest the possibility of identifying responder profiles, this study merely raises a hypothesis. Future studies incorporating formal interaction testing and stratified designs are needed to validate whether certain subgroups derive greater benefit from acupuncture. Such research may inform more personalized use of acupuncture and contribute to reducing the socioeconomic burden of HF.

RCS curve analysis of the number of acupuncture sessions indicated a decreasing trend in the aHRs for mortality due to circulatory system diseases and all-cause mortality for up to 30 sessions. However, after 30 sessions, a turning point was observed, and the trend showed an increase. In Korea, both inpatient and outpatient acupuncture treatments are covered by health insurance, allowing one outpatient and two inpatient sessions per day. Examination of the annual utilization of traditional Korean medicine outpatient services revealed an average of 9.4 visits per person annually (45). Acupuncture treatment is primarily administered through outpatient visits, with most patients seeking acupuncture during these visits, excluding those who receive counseling only (44, 49, 50). Therefore, patients who receive up to 100 acupuncture treatments per year are likely long-term inpatients. The change in the slope of the RCS curve with an increase in HR beyond 30 sessions may be attributed to the likelihood that inpatients have higher severity or suffer from chronic conditions than outpatients. Additionally, as the number of acupuncture sessions increased, the corresponding number of patients decreased, resulting in a nonsignificant CI of approximately 1 after around 100 sessions. A retrospective cohort study in Taiwan reported a decreasing HR for the occurrence of acute myocardial infarction in stroke patients receiving 15 or more acupuncture treatments, reaching 0.61. They observed a decreasing trend in the risk of occurrence with an increasing number of acupuncture treatment sessions (48). Furthermore, a systematic review comparing high- and low-dose acupuncture treatment groups reported that 37.5% of studies showed better responses in the high-dose group (51). These findings suggest that increasing the dosage of acupuncture treatment may enhance its therapeutic effects. Accordingly, this study also observed a significant trend in which higher exposure to acupuncture treatment was associated with a lower mortality rate in patients with HF. However, beyond 30 sessions, the trend showed a turning point with an increasing slope, indicating the need for additional research to explore whether the characteristics or severity of the disease in patients meeting the turning-point criteria changed. Additionally, further clinical studies are required to establish the clinically significant dosage of acupuncture treatment for patients with HF.

### 4.3 Outcome-influencing mechanisms

A retrospective cohort study of patients with HF who received a combination of TCM, including herbal medicine and acupuncture, reported a significant improvement in 5-year survival rates. In the compensated group, the aHR for mortality among TCM users was 0.14, indicating an 86% reduction. In the decompensated group—characterized by sudden or progressive worsening of HF symptoms—the aHR for mortality was 0.32, corresponding to a 68% reduction. These findings suggest a substantial survival benefit associated with TCM use in both clinical subgroups (29). Additionally, acupuncture treatment in ischemic stroke and hypertension patients showed a significantly reduced HR for all-cause mortality, with values of 0.32 and 0.73, respectively (52, 53). Excessive activation of the sympathetic nervous system is one of the major contributors to HF. Acupuncture has been reported to regulate sympathetic nervous activity while maintaining parasympathetic activity, leading to a reduction in heart rate (54, 55). Additionally, this regulatory mechanism is known to protect cardiomyocytes and reduce myocardial ischemia (26). These effects suggest that acupuncture shares some mechanisms with standard pharmacological treatments for HF and may have a clinically significant impact on HF management (56). Furthermore, acupuncture has been reported to exert cardiovascular benefits through various mechanisms (57), including reducing pro-inflammatory cytokine levels for anti-inflammatory effects (58, 59), increasing nitric oxide levels to improve endothelial function (60, 61), reducing oxidative stress (62) and modulating neurotransmitter (63). However, this study did not account for acupoints, as it relied on secondary data from insurance claims to the NHIS, making it challenging to ascertain the specific treatment acupoints administered to patients. Acupuncture is known to influence the long-loop pathway, including the hypothalamus, midbrain, and medulla, activating sensory nerves, spinal cord, and brain to alleviate organ dysfunction or inflammation through the autonomic nervous system (64–66). Although the existence of acupoint specificity has not been conclusively established, acupuncture is thought to exert therapeutic effects, even under untargeted conditions (64, 67). This suggests that acupuncture at specific acupoints goes beyond local regulation and has a systemic effect on various body systems. Furthermore, the diverse treatment procedures in traditional medicine, in which the same prescription may be used for different conditions or different prescriptions for the same condition, impose limitations on the application of RWD research by restricting the acupoints or primary diseases targeted for acupuncture.

### 4.4 Strengths and limitations

To the best of our knowledge, this is the first retrospective cohort study to investigate the association between acupuncture treatment and reduced mortality in patients with HF. It overcomes the limitations highlighted in previous observational studies on HF by conducting long-term tracking, confirming mortality as a clinical outcome, and performing dose-response analysis with both continuous and nominal categories (31). However, this study had several limitations. First, this study assessed only exposure to acupuncture treatment without restricting specific acupoints or diseases. Since claims data are primarily collected for insurance reimbursement, they lack information on treatment protocols, session frequency, or duration. Additionally, this study conducted a survival analysis based on Korean data, and because treatment procedures may vary by country, universal application of the results may be restricted. Therefore, studies based on observational data from other countries are required to achieve a broader applicability. Second, the severity of HF and some risk factors were not adjusted for, which is a limitation of using secondary data. The severity of HF cannot be confirmed when patients are included based on disease codes. Additionally, risk factors for heart disease, such as smoking, body mass index, and physical activity, can only be confirmed from health checkup data. However, since not all citizens undergo health checkups, including these factors as covariates raises concerns about sample size reduction, leading to their exclusion from a study involving a large sample size. Therefore, future research should utilize a customized database to study the entire patient population and incorporate health checkup data into the analysis. Furthermore, to more accurately assess and adjust for HF severity and risk factors, additional studies should be conducted by linking hospital records with NHIS data. Third, potential confounders may exist between patients using acupuncture and those not utilizing it, factors that we may not be aware of. This concern has been underscored in the patient characteristics of this study and has been mentioned in previous studies using RWD in Korea, highlighting the imbalance between the AT and non-AT groups. However, a commonality was observed in the AT group, where patients with more comorbidities and severity were often included (36, 52, 53). Therefore, future epidemiological studies are necessary to investigate whether differences in severity or healthcare utilization patterns exist between groups using and not using traditional Korean medicine. In the present analysis, we addressed intergroup heterogeneity using PS matching to control for known confounders. While HRs were calculated based on the matched dataset, we additionally reported aHRs controlling for the same covariates used in the PS matching. Although this raises the possibility of overadjustment due to redundant covariate control, the differences between PS-matched and aHRs were minimal across both overall and dose-response analyses. This consistency suggests that the observed associations were robust and not overly sensitive to the modeling approach. Fourth**,** we did not implement a washout period for acupuncture exposure and conducted a study focusing on prevalent users. Observational studies suggest that existing users tend to exhibit better outcomes than new users (68). Although the duration of acupuncture effectiveness is unknown, rigorous verification of the association between acupuncture exposure and reduced mortality requires a washout period. Fifth, it is uncertain whether 1 year after the diagnosis of HF is an appropriate landmark time point. Time-dependent models and landmark methodologies are commonly employed to eliminate the immortal time bias in observational studies, with time-dependent models considered the gold standard (69, 70). In this study, the analysis was conducted based on previously established landmark time points, but sensitivity analysis was not performed to examine how mortality hazards vary depending on the landmark time point. Nevertheless, longer landmark times tend to reduce bias and decrease the likelihood of overestimation (35, 69, 71), suggesting that applying a one-year landmark in this study is a reasonable choice. However, to enhance the rigor of the results, additional statistical analyses are necessary. In particular, sensitivity analyses should be conducted on the definition of acupuncture exposure and the index date, which were arbitrarily determined by the research team. Therefore, future studies will incorporate additional statistical models and conduct sensitivity analyses on these predefined variables.

## Data Availability

Publicly available datasets were analyzed in this study. This data can be found here: We utilized the sample cohort database from https://nhiss.nhis.or.kr, and the access number is NHIS-2022-2-205.

## References

[1] Bozkurt B Coats AJ Tsutsui H Abdelhamid M Adamopoulos S Albert N Universal definition and classification of heart failure: a report of the heart failure society of America, heart failure association of the European Society of Cardiology, Japanese heart failure society and writing committee of the universal definition of heart failure. J Card Fail. (2021) S1071-9164(21):00050–6. 10.1016/j.cardfail.2021.01.022 33663906

[2] Savarese G Becher PM Lund LH Seferovic P Rosano GMC Coats AJS. Global burden of heart failure: a comprehensive and updated review of epidemiology. Cardiovasc Res. (2023) 118:3272–87. 10.1093/cvr/cvac013 35150240

[3] Bragazzi NL Zhong W Shu J Abu Much A Lotan D Grupper A Burden of heart failure and underlying causes in 195 countries and territories from 1990 to 2017. Eur J Prev Cardiol. (2021) 28:1682–90. 10.1093/eurjpc/zwaa147 33571994

[4] The Korean Society of Heart Failure. 2022 *KSHF Guideline for the Management of HEART FAILURE*. (2022).

[5] Emmons-Bell S Johnson C Roth G. Prevalence, incidence and survival of heart failure: a systematic review. Heart Br Card Soc. (2022) 108:1351–60. 10.1136/heartjnl-2021-320131 PMC9380485 35042750

[6] Shah KS Xu H Matsouaka RA Bhatt DL Heidenreich PA Hernandez AF Heart failure with preserved, borderline, and reduced ejection fraction: 5-year outcomes. J Am Coll Cardiol. (2017) 70:2476–86. 10.1016/j.jacc.2017.08.074 29141781

[7] Jones NR Roalfe AK Adoki I Hobbs FDR Taylor CJ. Survival of patients with chronic heart failure in the community: a systematic review and meta-analysis. Eur J Heart Fail. (2019) 21:1306–25. 10.1002/ejhf.1594 31523902 PMC6919428

[8] Rosano GMC Moura B Metra M Böhm M Bauersachs J Ben Gal T Patient profiling in heart failure for tailoring medical therapy. A consensus document of the heart failure association of the European Society of Cardiology. Eur J Heart Fail. (2021) 23:872–81. 10.1002/ejhf.2206 33932268

[9] Heidenreich PA Bozkurt B Aguilar D Allen LA Byun JJ Colvin MM 2022 AHA/ACC/HFSA guideline for the management of heart failure: executive summary: a report of the American College of Cardiology/American Heart Association joint committee on clinical practice guidelines. J Am Coll Cardiol. (2022) 79:1757–80. 10.1016/j.jacc.2021.12.011 35379504

[10] Savarese G Kishi T Vardeny O Adamsson Eryd S Bodegård J Lund LH Heart failure drug treatment-inertia, titration, and discontinuation: a multinational observational study (EVOLUTION HF). JACC Heart Fail. (2023) 11:1–14. 10.1016/j.jchf.2022.08.009 36202739

[11] Ahmed A Fonarow GC Zhang Y Sanders PW Allman RM Arnett DK Renin-angiotensin inhibition in systolic heart failure and chronic kidney disease. Am J Med. (2012) 125:399–410. 10.1016/j.amjmed.2011.10.013 22321760 PMC3324926

[12] Rich MW. Pharmacotherapy of heart failure in the elderly: adverse events. Heart Fail Rev. (2012) 17:589–95. 10.1007/s10741-011-9263-1 21688185

[13] Crespo-Leiro MG Barge-Caballero E Segovia-Cubero J González-Costello J López-Fernández S García-Pinilla JM Hyperkalemia in heart failure patients in Spain and its impact on guidelines and recommendations: ESC-EORP-HFA heart failure long-term registry. Rev Espanola Cardiol Engl Ed. (2020) 73:313–23. 10.1016/j.rec.2019.05.015 31672562

[14] Maggioni AP Anker SD Dahlström U Filippatos G Ponikowski P Zannad F Are hospitalized or ambulatory patients with heart failure treated in accordance with European society of cardiology guidelines? Evidence from 12,440 patients of the ESC heart failure long-term registry. Eur J Heart Fail. (2013) 15:1173–84. 10.1093/eurjhf/hft134 23978433

[15] Trevisan M de Deco P Xu H Evans M Lindholm B Bellocco R Incidence, predictors and clinical management of hyperkalaemia in new users of mineralocorticoid receptor antagonists. Eur J Heart Fail. (2018) 20:1217–26. 10.1002/ejhf.1199 29667759 PMC6607478

[16] Du Y Duan C Yang Y Yuan G Zhou Y Zhu X Heart transplantation: a bibliometric review from 1990 to 2021. Curr Probl Cardiol. (2022) 47:101176. 10.1016/j.cpcardiol.2022.101176 35341797

[17] Liang B Gu N. Traditional Chinese medicine for coronary artery disease treatment: clinical evidence from randomized controlled trials. Front Cardiovasc Med. (2021) 8:702110. 10.3389/fcvm.2021.702110 34422929 PMC8377193

[18] Jin H Kang S Park D Mun Y-J Leem J. Effectiveness and safety of Liriope tuber (liriopis seu ophiopogonis tuber, maidong) included traditional herbal medicine for patients with paroxysmal atrial fibrillation: a systematic review, meta-analysis, and network analysis. Integr Med Res. (2024) 13:101069. 10.1016/j.imr.2024.101069 39247398 PMC11378115

[19] Ahn J-Y Chu H Leem J Yun J-M. Effectiveness and safety of traditional herbal medicine on cardiac arrhythmic condition: a systematic review and meta-analysis of randomized control clinical trial. Medicine. (2024) 103:e38441. 10.1097/MD.0000000000038441 38847675 PMC11155608

[20] Jun H Park D Sul J-U Cheong MJ Kim H Youn I Impact of acupuncture on mortality in patients with disabilities and newly diagnosed heart failure: a nationwide cohort study. Front Med. (2025) 12:1519588. 10.3389/fmed.2025.1519588 PMC11813762 39944496

[21] Yun S Oh J Chu H Park D Leem J. Systematic review of preclinical studies on the efficacy and mechanisms of herbal medicines in post-myocardial infarction heart failure with reduced ejection fraction. Med Kaunas Lith. (2024) 60:1101. 10.3390/medicina60071101 PMC11278938 39064530

[22] Jeong SH Lee H-G Kim G Kwon S Cho S-Y Jung W-S Combination therapy of acupuncture and herbal medicine for heart failure: a systematic review and meta-analysis. Medicine. (2024) 103:e39061. 10.1097/MD.0000000000039061 39093749 PMC11296463

[23] Chow SL Bozkurt B Baker WL Bleske BE Breathett K Fonarow GC Complementary and alternative medicines in the management of heart failure: a scientific statement from the American Heart Association. Circulation. (2023) 147:e4–e30. 10.1161/CIR.0000000000001110 36475715

[24] Middlekauff HR Hui K Yu JL Hamilton MA Fonarow GC Moriguchi J Acupuncture inhibits sympathetic activation during mental stress in advanced heart failure patients. J Card Fail. (2002) 8:399–406. 10.1054/jcaf.2002.129656 12528093

[25] Kristen AV Schuhmacher B Strych K Lossnitzer D Friederich H-C Hilbel T Acupuncture improves exercise tolerance of patients with heart failure: a placebo-controlled pilot study. Heart Br Card Soc. (2010) 96:1396–400. 10.1136/hrt.2009.187930 20554511

[26] Ni Y-M Frishman WH. Acupuncture and cardiovascular disease: focus on heart failure. Cardiol Rev. (2018) 26:93–8. 10.1097/CRD.0000000000000179 29419562

[27] Lee H Kim T-H Leem J. Acupuncture for heart failure: a systematic review of clinical studies. Int J Cardiol. (2016) 222:321–31. 10.1016/j.ijcard.2016.07.195 27500758

[28] Liang B Yan C Zhang L Yang Z Wang L Xian S The effect of acupuncture and moxibustion on heart function in heart failure patients: a systematic review and meta-analysis. Evid-Based Complement Altern Med ECAM. (2019) 2019:6074967. 10.1155/2019/6074967 PMC6854931 31772597

[29] Tsai M-Y Hu W-L Chiang J-H Huang Y-C Chen S-Y Hung Y-C Improved medical expenditure and survival with integration of traditional Chinese medicine treatment in patients with heart failure: a nationwide population-based cohort study. Oncotarget. (2017) 8:90465–76. 10.18632/oncotarget.20063 29163845 PMC5685766

[30] Kim D Kwon SH Chung SH Ahn BR Lim B-M. The health insurance system and the quality improvement policies for Chinese medicine in Taiwan. Soc Prev Korean Med. (2016) 20:27–38.

[31] Park J Bak S Chu H Kang S Youn I Jun H Current research Status and implication for further study of real-world data on east Asian traditional medicine for heart failure: a scoping review. Healthcare. (2024) 12:61. 10.3390/healthcare12010061 PMC10779411 38200969

[32] Schneeweiss S Rassen JA Brown JS Rothman KJ Happe L Arlett P Graphical depiction of longitudinal study designs in health care databases. Ann Intern Med. (2019) 170:398–406. 10.7326/M18-3079 30856654

[33] Eligible Beneficiaries and Population for Health Insurance. NHIS. Available online at: http://www.nhis.or.kr/nhis/policy/wbhada01700m01.do (Accessed April 25, 2024).

[34] Lee J Lee JS Park S-H Shin SA Kim K. Cohort profile: the national health insurance service-national sample cohort (NHIS-NSC), South Korea. Int J Epidemiol. (2017) 46:e15. 10.1093/ije/dyv319 26822938

[35] Mi X Hammill BG Curtis LH Lai EC-C Setoguchi S. Use of the landmark method to address immortal person-time bias in comparative effectiveness research: a simulation study. Stat Med. (2016) 35:4824–36. 10.1002/sim.7019 27350312

[36] Jung H Won T Kim G-Y Jang J Yeo S Lim S. Efficacy of acupuncture on cardiovascular complications in patients with diabetes mellitus in Korea: a nationwide retrospective cohort. J Integr Med. (2023) 21:176–83. 10.1016/j.joim.2023.01.007 36797171

[37] Jeong S. Association of Atrial Fibrillation Patients’ Exposure to Acupuncture Treatments and Risk of Stroke and Mortality : A Nationwide Retrospective Cohort Study in Korea. Iksan, Jeollabuk-do: Wonkwang University (2022).

[38] D’Errico MM Piscitelli P Mirijello A Santoliquido M Salvatori M Vigna C CHA2DS2-VASc And R2CHA2DS2-VASc scores predict mortality in high cardiovascular risk population. Eur J Clin Invest. (2022) 52:e13830. 10.1111/eci.13830 35778894

[39] Chan Y-H Yiu K-H Lau K-K Yiu Y-F Li S-W Lam T-H The CHADS2 and CHA2DS2-VASc scores predict adverse vascular function, ischemic stroke and cardiovascular death in high-risk patients without atrial fibrillation: role of incorporating PR prolongation. Atherosclerosis. (2014) 237:504–13. 10.1016/j.atherosclerosis.2014.08.026 25463082

[40] Quan H Sundararajan V Halfon P Fong A Burnand B Luthi J-C Coding algorithms for defining comorbidities in ICD-9-CM and ICD-10 administrative data. Med Care. (2005) 43:1130–9. 10.1097/01.mlr.0000182534.19832.83 16224307

[41] Quan H Li B Couris CM Fushimi K Graham P Hider P Updating and validating the Charlson comorbidity index and score for risk adjustment in hospital discharge abstracts using data from 6 countries. Am J Epidemiol. (2011) 173:676–82. 10.1093/aje/kwq433 21330339

[42] Killick R Fearnhead P Eckley IA. Optimal detection of changepoints with a linear computational cost. J Am Stat Assoc. (2012) 107:1590–8. 10.1080/01621459.2012.737745

[43] Goepp V Bouaziz O Nuel G. Spline Regression with Automatic Knot Selection. *arXiv.org* (2018). 10.48550/arXiv.1808.01770.

[44] Lee S Yeo J Lee S-H Lee YJ Lee S Ha I-H. Trends in healthcare utilisation of patients with migraine in South Korea: a retrospective observational study using health insurance review and assessment service national patient sample data from 2010 to 2018. BMJ Open. (2023) 13:e059926. 10.1136/bmjopen-2021-059926 36944456 PMC10032417

[45] KOrean Statistical Infromation Service. Number of Traditional Korean Medicine Outpatient Clinic Visits in the Past Year. Available online at: https://kosis.kr/statHtml/statHtml.do?orgId=117&tblId=DT_117087N_001&vw_cd=MT_ZTITLE&list_id=117_11787_A04_001&scrId=&seqNo=&lang_mode=ko&obj_var_id=&itm_id=&conn_path=MT_ZTITLE&path=%252FstatisticsList%252FstatisticsListIndex.do (Accessed November 15, 2023).

[46] Witt CM Schützler L Lüdtke R Wegscheider K Willich SN. Patient characteristics and variation in treatment outcomes: which patients benefit most from acupuncture for chronic pain? Clin J Pain. (2011) 27:550–5. 10.1097/AJP.0b013e31820dfbf5 21317771

[47] Witt CM Vertosick EA Foster NE Lewith G Linde K MacPherson H The effect of patient characteristics on acupuncture treatment outcomes: an individual patient data meta-analysis of 20,827 chronic pain patients in randomized controlled trials. Clin J Pain. (2019) 35:428–34. 10.1097/AJP.0000000000000691 30908336 PMC6450709

[48] Chuang S-F Shih C-C Yeh C-C Lane H-L Tsai C-C Chen T-L Decreased risk of acute myocardial infarction in stroke patients receiving acupuncture treatment: a nationwide matched retrospective cohort study. BMC Complement Altern Med. (2015) 15:318. 10.1186/s12906-015-0828-8 26353964 PMC4563856

[49] Son C Lim Y-C Lee Y-S Lim J-H Kim B-K Ha I-H. Analysis of medical services for insomnia in Korea: a retrospective, cross-sectional study using the health insurance review and assessment claims data. Healthcare. (2022) 10:7. 10.3390/healthcare10010007 PMC8775632 35052172

[50] Ahn J Yeo J Lee S-H Lee YJ Park Y Goo B Healthcare usage and cost for plantar fasciitis: a retrospective observational analysis of the 2010–2018 health insurance review and assessment service national patient sample data. BMC Health Serv Res. (2023) 23:546. 10.1186/s12913-023-09443-2 37231457 PMC10210451

[51] Yoon D-E Lee I-S Chae Y. Identifying dose components of manual acupuncture to determine the dose-response relationship of acupuncture treatment: a systematic review. Am J Chin Med. (2022) 50:653–71. 10.1142/S0192415X22500264 35300569

[52] Choi S-R Kim E-S Jang B-H Jung B Ha I-H. A time-dependent analysis of association between acupuncture utilization and the prognosis of ischemic stroke. Healthc Basel Switz. (2022) 10:756. 10.3390/healthcare10050756 PMC9141209 35627893

[53] Jung H Yeo S Lim S. Effects of acupuncture on cardiovascular risks in patients with hypertension: a Korean cohort study. Acupunct Med. (2021) 39:116–25. 10.1177/0964528420920290 32567334

[54] Middlekauff HR. Acupuncture in the treatment of heart failure. Cardiol Rev. (2004) 12:171. 10.1097/01.crd.0000103650.71735.f0 15078586

[55] Hamvas S Hegyi P Kiss S Lohner S McQueen D Havasi M. Acupuncture increases parasympathetic tone, modulating HRV—systematic review and meta-analysis. Complement Ther Med. (2023) 72:102905. 10.1016/j.ctim.2022.102905 36494036

[56] Zhang DY Anderson AS. The sympathetic nervous system and heart failure. Cardiol Clin. (2014) 32:33–45, vii. 10.1016/j.ccl.2013.09.010 24286577 PMC5873965

[57] Wang S Fang R Huang L Zhou L Liu H Cai M Acupuncture in traditional Chinese medicine: a complementary approach for cardiovascular health. J Multidiscip Healthc. (2024) 17:3459–73. 10.2147/JMDH.S476319 39050695 PMC11268752

[58] Liaw JJT Peplow PV. Effect of electroacupuncture on inflammation in the obese zucker fatty rat model of metabolic syndrome. J Acupunct Meridian Stud. (2016) 9:73–9. 10.1016/j.jams.2015.08.004 27079228

[59] Liaw JJT Peplow PV. Differential effect of electroacupuncture on inflammatory adipokines in two rat models of obesity. J Acupunct Meridian Stud. (2016) 9:183–90. 10.1016/j.jams.2016.02.002 27555223

[60] Tsuchiya M Sato EF Inoue M Asada A. Acupuncture enhances generation of nitric oxide and increases local circulation. Anesth Analg. (2007) 104:301–7. 10.1213/01.ane.0000230622.16367.fb 17242084

[61] Ma S-X Lee PC Anderson TL Li X-Y Jiang IZ. Response of local nitric oxide release to manual acupuncture and electrical heat in humans: effects of reinforcement methods. Evid-Based Complement Altern Med ECAM. (2017) 2017:4694238. 10.1155/2017/4694238 PMC5498898 28717380

[62] Zhao Y Zhou B Zhang G Xu S Yang J Deng S The effect of acupuncture on oxidative stress: a systematic review and meta-analysis of animal models. PLoS One. (2022) 17:e0271098. 10.1371/journal.pone.0271098 36084019 PMC9462787

[63] Li P Longhurst JC. Neural mechanism of electroacupuncture’s hypotensive effects. Auton Neurosci. (2010) 157:24–30. 10.1016/j.autneu.2010.03.015 20444652 PMC2947600

[64] Longhurst J. Acupuncture’s cardiovascular actions: a mechanistic perspective. Med Acupunct. (2013) 25:101–13. 10.1089/acu.2013.0960 24761168 PMC3616410

[65] Li P Tjen-A-Looi SC Guo Z-L Fu L-W Longhurst JC. Long-loop pathways in cardiovascular electroacupuncture responses. J Appl Physiol Bethesda Md 1985. (2009) 106:620–30. 10.1152/japplphysiol.91277.2008 PMC2644252 19074569

[66] Li Y-W Li W Wang S-T Gong Y-N Dou B-M Lyu Z-X The autonomic nervous system: a potential link to the efficacy of acupuncture. Front Neurosci. (2022) 16:1038945. 10.3389/fnins.2022.1038945 36570846 PMC9772996

[67] Zhang H Bian Z Lin Z. Are acupoints specific for diseases? A systematic review of the randomized controlled trials with sham acupuncture controls. Chin Med. (2010) 5:1. 10.1186/1749-8546-5-1 20145733 PMC2818640

[68] Danaei G Tavakkoli M Hernán MA. Bias in observational studies of prevalent users: lessons for comparative effectiveness research from a meta-analysis of statins. Am J Epidemiol. (2012) 175:250–62. 10.1093/aje/kwr301 22223710 PMC3271813

[69] Zheng Q Otahal P Cox IA de Graaff B Campbell JA Ahmad H The influence of immortal time bias in observational studies examining associations of antifibrotic therapy with survival in idiopathic pulmonary fibrosis: a simulation study. Front Med. (2023) 10:1157706. 10.3389/fmed.2023.1157706 PMC10126672 37113607

[70] Vail EA Gershengorn HB Wunsch H Walkey AJ. Attention to immortal time bias in critical care research. Am J Respir Crit Care Med. (2021) 203:1222–9. 10.1164/rccm.202008-3238CP 33761299

[71] Mi X Hammill BG Curtis LH Greiner MA Setoguchi S. Impact of immortal person-time and time scale in comparative effectiveness research for medical devices: a case for implantable cardioverter-defibrillators. J Clin Epidemiol. (2013) 66:S138–44. 10.1016/j.jclinepi.2013.01.014 23849148

