# Supplementary material for: Association between acupuncture treatment exposure and mortality in patients with heart failure: a nationwide cohort study
Source: Front Cardiovasc Med. 2025 Jul 15;12:1461302. doi: 10.3389/fcvm.2025.1461302 (PMC12305702; doi:10.3389/fcvm.2025.1461302)
Supplement: Supplementary file 1 [file Datasheet1.pdf]

Supplementary Figure S1. Hazard ratio changes with dose of acupuncture treatment exposure

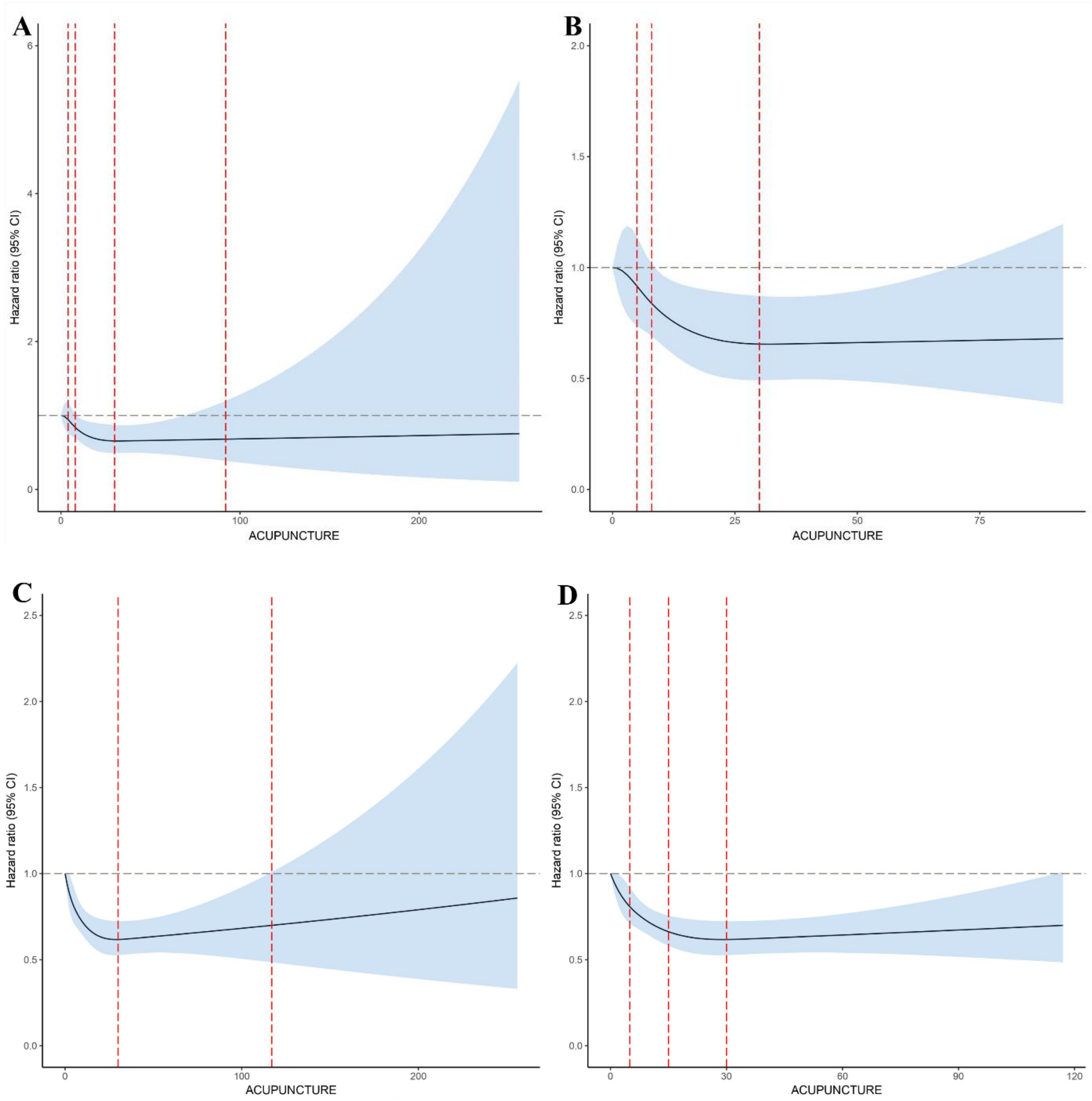

The red line represents the inflection point of the hazard ratio slope. Hazard ratios were adjusted for age, sex, residential area, income level, disability grade, CHA_2DS_2-VASc score, Charlson Comorbidity Index, and comorbidities (hypertension, diabetes mellitus, dyslipidemia, myocardial infarction, peripheral artery disease, chronic obstructive pulmonary disease, and cancer).

(A), (B) Circulatory system disease-related mortality and (C), (D) all-cause mortality.

Supplementary Figure S2. Forest plot of subgroup analysis

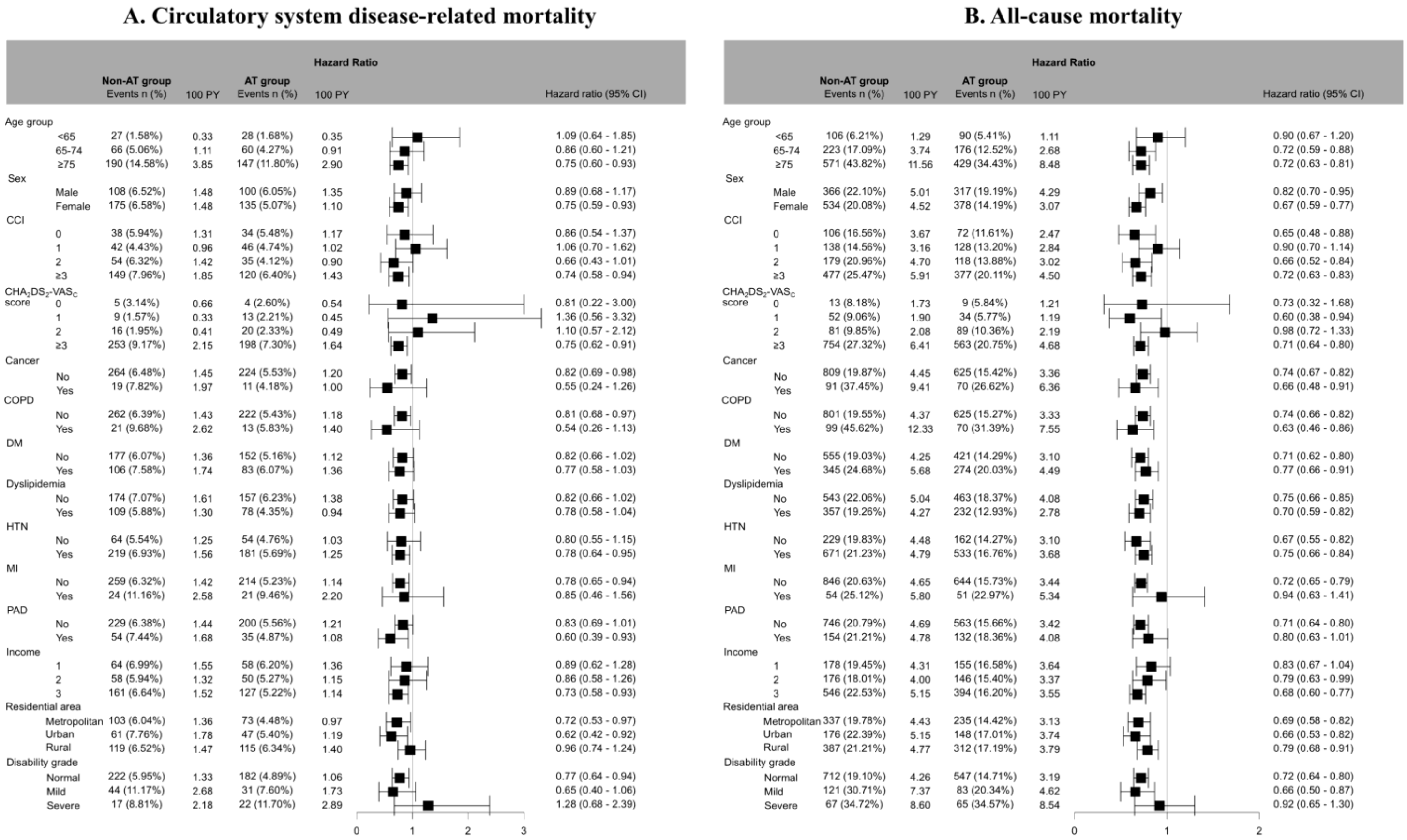

AT group, patients with acupuncture exposure ≥2 times within the first year post-diagnosis; CCI, Charlson comorbidity index; CI, confidence interval; COPD, chronic obstructive pulmonary disease; DM, diabetes mellitus; HTN, hypertension; MI, myocardial infarction; Non-AT group, subjects not exposed to acupuncture within the first year after diagnosis; PAD, peripheral artery disease, PY, person-year.

Hazard ratios were estimated using Cox proportional hazards models. All models were adjusted for age, sex, residential area, income level, disability grade, CHA_2DS_2-VASc score, Charlson Comorbidity Index, and comorbidities (hypertension, diabetes mellitus, dyslipidemia, myocardial infarction, peripheral artery disease, chronic obstructive pulmonary disease, and cancer), excluding the stratification variable in each subgroup.

Supplementary Figure S3. Flowchart of sample selection in sensitivity analysis

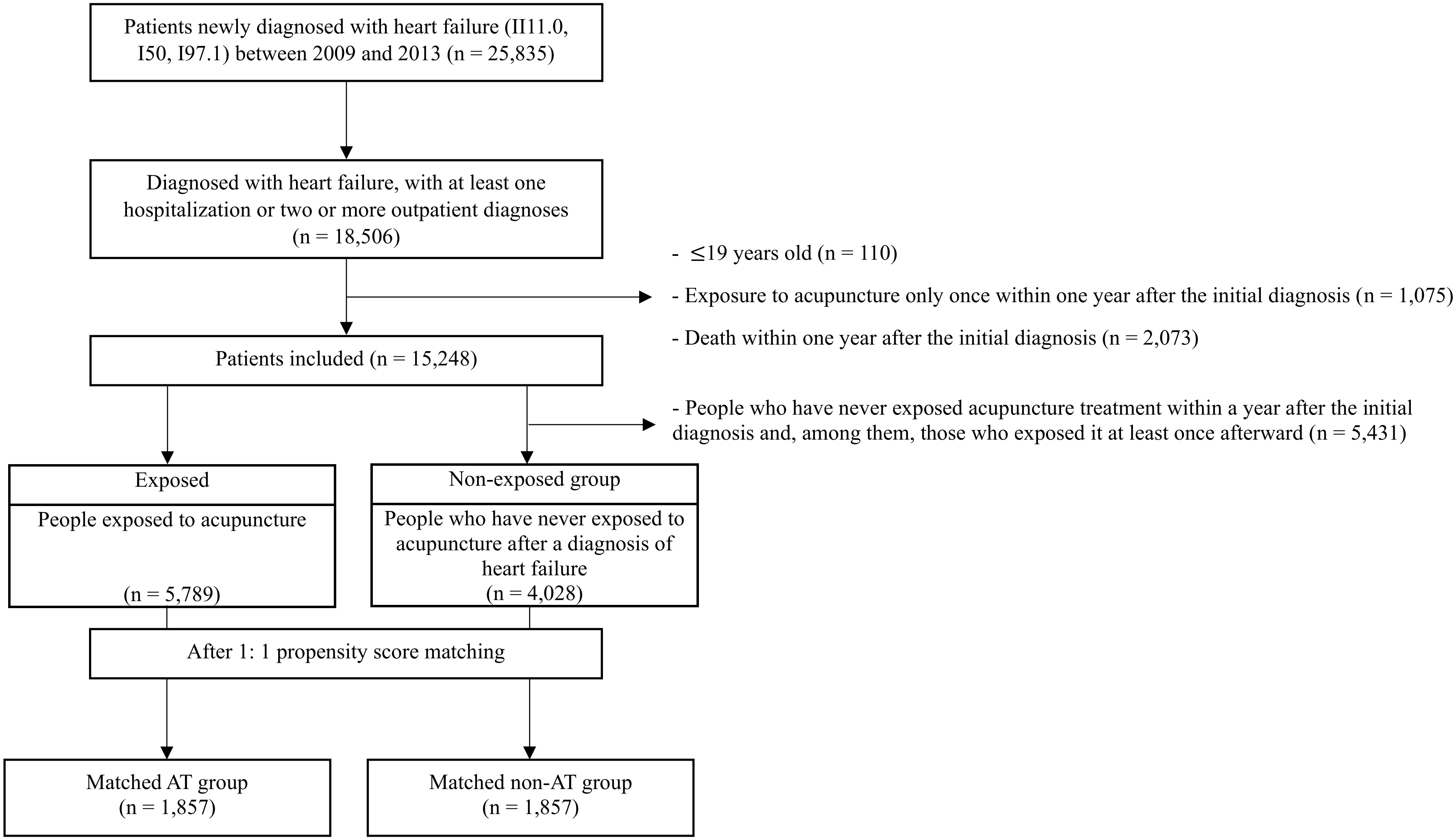

AT, acupuncture treatment group; non-AT, non-acupuncture treatment group

Supplementary Figure S4. Kaplan–Meier curves in sensitivity analysis

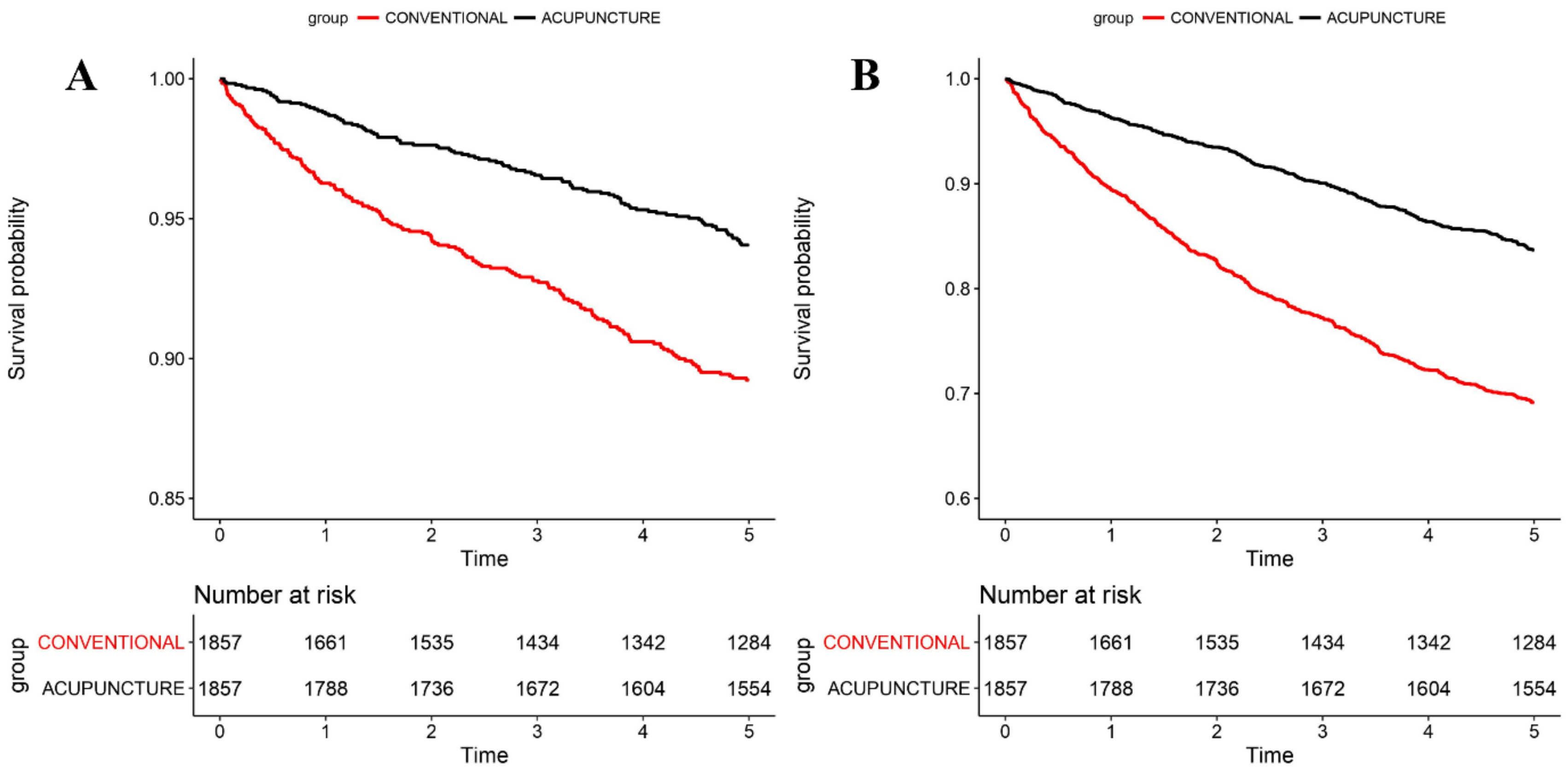

Kaplan-Meier curve showing the survival probability in the acupuncture and non-acupuncture treatment groups after PS matching in the sensitivity analysis. Both A and B show p-values <0.05, indicating a significant difference. (A) Circulatory system disease-related mortality and (B) all-cause mortality.

Supplementary Figure S5. Kaplan–Meier curves of dose-response in sensitivity analysis

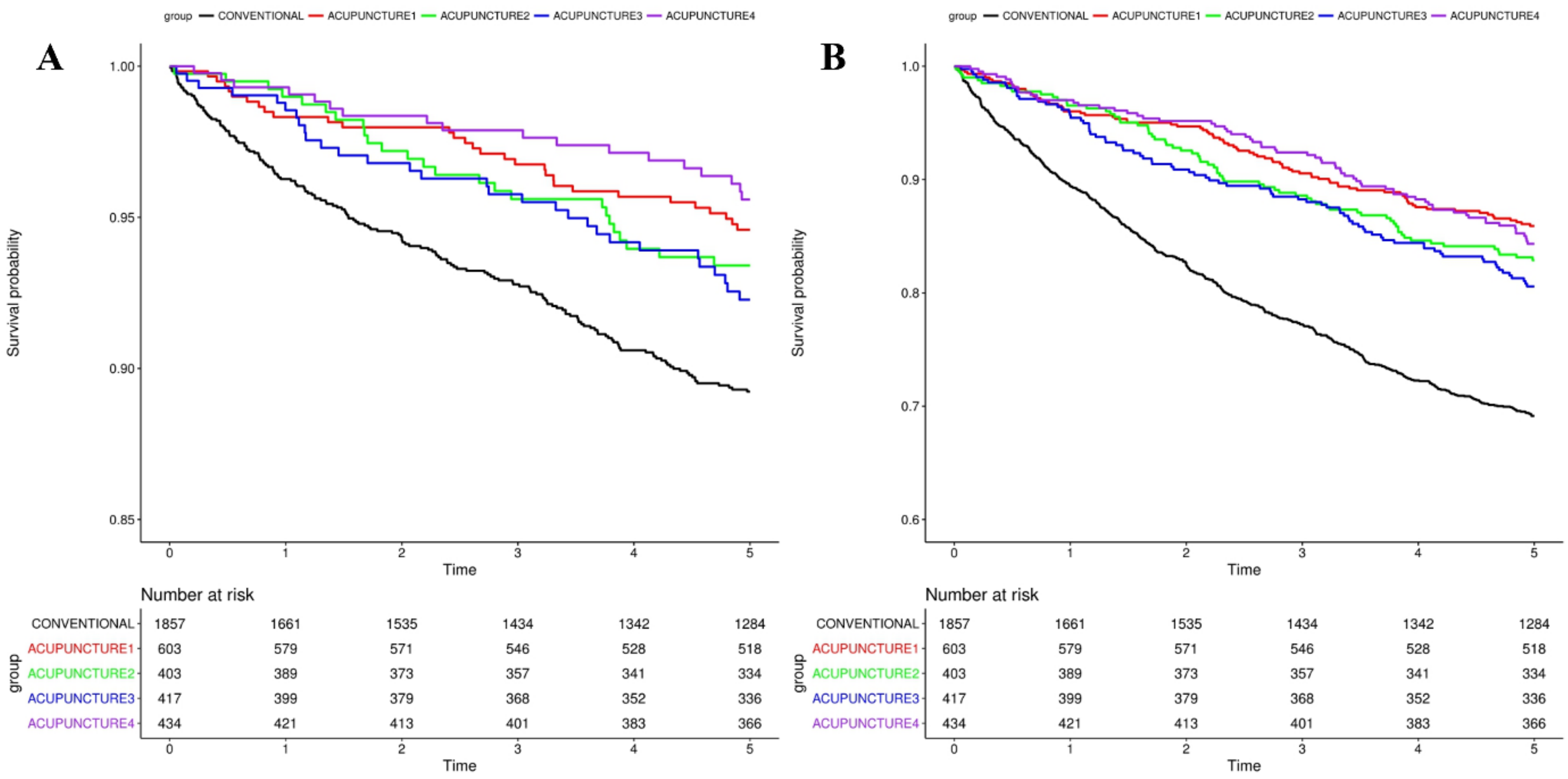

Kaplan–Meier curve showing the survival probability in the four groups, stratified by the number of acupuncture treatments, along with the non-acupuncture treatment group after PS matching in the sensitivity analysis. Both A and B show p-values <0.05, indicating a significant difference. (A) Circulatory system disease-related mortality and (B) all-cause mortality.

Supplementary Figure S6. Hazard ratio changes with dose of acupuncture treatment exposure in sensitivity analysis

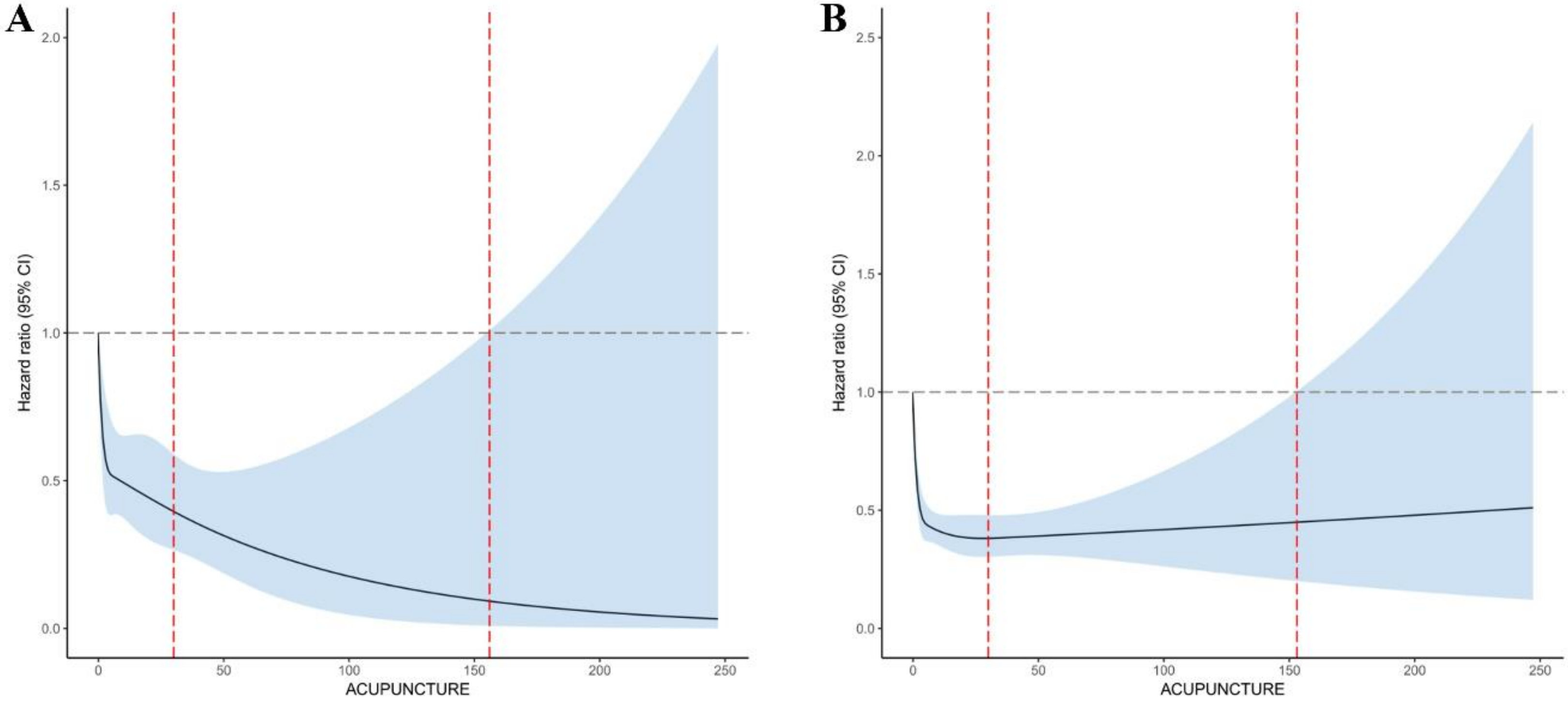

The red line represents the inflection point of the hazard ratio slope. Hazard ratios were adjusted for age, sex, residential area, income level, disability grade, CHA_2DS_2-VASc score, Charlson Comorbidity Index, and comorbidities (hypertension, diabetes mellitus, dyslipidemia, myocardial infarction, peripheral artery disease, chronic obstructive pulmonary disease, and cancer).

(A) Circulatory system disease-related mortality and (B) all-cause mortality.

Supplementary Figure S7. Forest plot of subgroup in sensitivity analysis

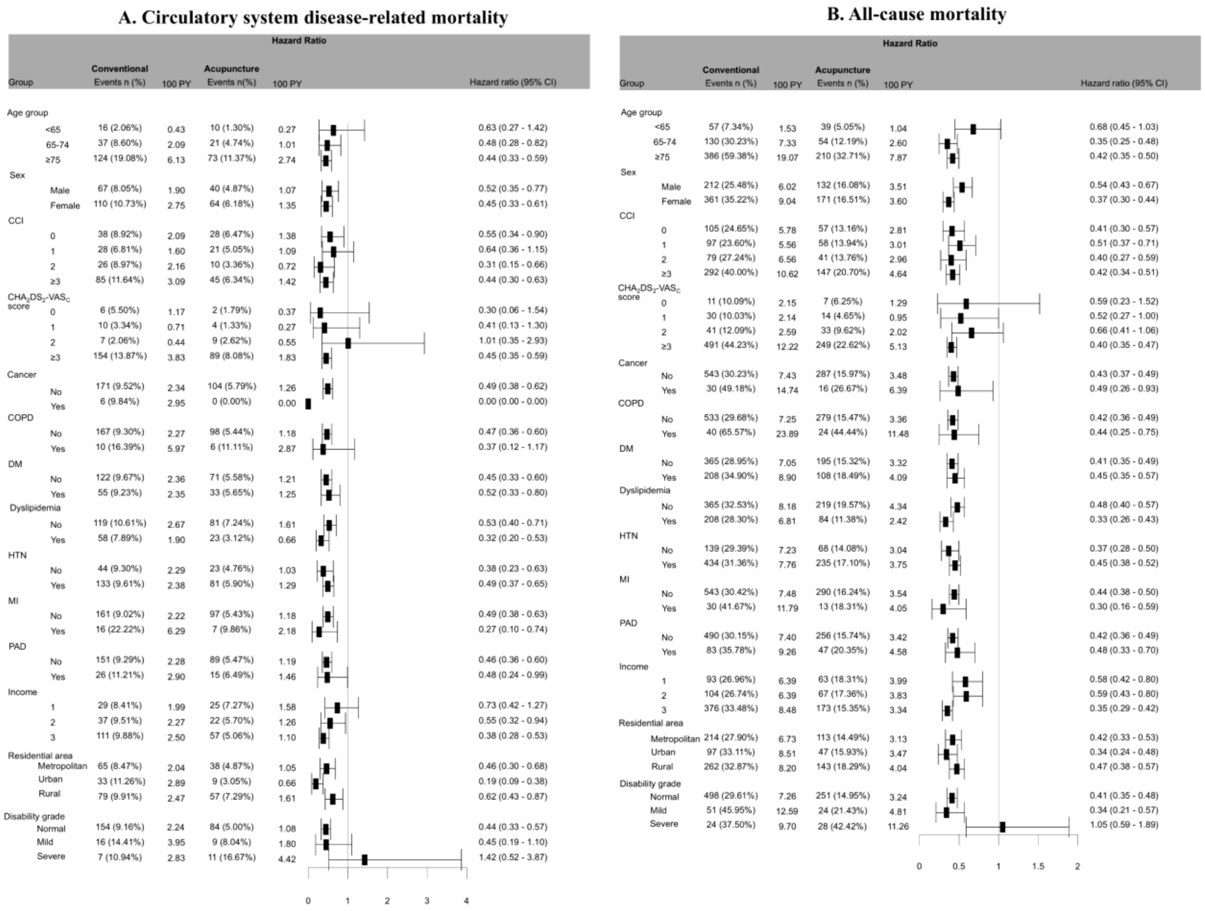

AT group, patients with acupuncture exposure ≥2 times within the first year post-diagnosis; CCI, Charlson comorbidity index; CI, confidence interval; COPD, chronic obstructive pulmonary disease; DM, diabetes mellitus; HTN, hypertension; MI, myocardial infarction; Non-AT group, patients who have never been exposed to acupuncture after diagnosis; PAD, peripheral artery disease, PY, person-year.

Hazard ratios were estimated using Cox proportional hazards models. All models were adjusted for age, sex, residential area, income level, disability grade, CHA_2DS_2-VASc score, Charlson Comorbidity Index, and comorbidities (hypertension, diabetes mellitus, dyslipidemia, myocardial infarction, peripheral artery disease, chronic obstructive pulmonary disease, and cancer), excluding the stratification variable in each subgroup.

Supplementary Table S1. Definitions of comorbidities

Covariates	Category	Definition
Patient characteristics		
Income	1) Medical aid (0 in decile) & Low (1,2,3 in decile)	1) Income lowest 30%
	2) Middle (4,5,6,7 in decile)	2) Income middle range 40%
	3) High (8,9,10 in decile in decile)	3) Income highest 30%
Residential area	Metropolitan	The Greater Seoul Metropolitan Area (Seoul, Incheon, Gyunggi); Metropolitan area of Republic of Korea. 26 million, more than 50% of the total population
	Urban	Daejeon, Gwangju, Ulsan, Pusan, Daegu
	Rural	Others
Disability grade	Non-disabled	Non-disability
	Mild	Grade 3 to 6
	Severe	Grade 1 to 2
Comorbidities	ICD-10 code	
Hypertension	I10, I11, I12, I13, I15	Admission≥1 or outpatient department≥2
Diabetes mellitus	E10–E14	Admission≥1 or outpatient department≥2
Dyslipidemia	E78	Admission≥1 or outpatient department≥2
Myocardial infarction	I21, I22, I252	Admission≥1 or outpatient department≥2
Peripheral artery disease	I70, I73	Admission≥1 or outpatient department≥2
Chronic Obstructive Pulmonary Disease	J41–44	Admission≥1 or outpatient department≥2
Cancer	C00–C97 and RID code (V193)	Admission≥1 or outpatient department≥2
Risk score		
CHA_2DS_2-VASc Score	0, 1, 2, ≥3	Heart failure (1 point), hypertension (1 point), age ≥75 years (2 points), diabetes mellitus (1 point), previous stroke/systemic embolism/transient ischemic attack (2 points), vascular disease (prior myocardial infarction or peripheral artery disease, 1 point), age 65–74 years (1 point) and female sex (1 point)
Charlson comorbidity index	0, 1, 2, ≥3	Age between 41 and 50 (1 point), between 51 and 60 (2 points), between 61 and 70 (3 points), 71 years of age or older (4 points) Myocardial infarction, congestive heart failure, peripheral vascular disease, dementia, cerebrovascular disease, chronic pulmonary disease, connective tissue disease, ulcer, chronic liver disease, diabetes mellitus (1 point)

	Hemiplegia, moderate to severe kidney disease, diabetes mellitus with end organ damage, solid tumor, leukemia, lymphoma (2 points)<br>Moderate to severe liver disease (3 points)<br>Metastatic solid tumor, AIDS (6 points)

AIDS, Acquired Immune deficiency Syndrome; ICD-10, 10th revision of the International Classification of Diseases.

Supplementary Table S2. Hazard ratios based on the number of acupuncture treatments for circulatory system disease-related mortality

	Total, N	Event, n (%)	IR (per 100PY)	PS-matched HR (95% CI)	P	Adjusted HR (95% CI)*	P
Non-AT group	4315	283 (6.56)	1.48	1 [Ref]		1 [Ref]	
AT group							
≥2	4315	235 (5.45)	1.19	0.81 (0.68–0.96)	0.02	0.79 (0.67–0.94)	0.01
≥3	3761	209 (5.56)	1.22	0.82 (0.69–0.99)	0.03	0.80 (0.66–0.95)	0.01
≥4	3290	180 (5.47)	1.2	0.81 (0.67–0.98)	0.03	0.77 (0.64–0.93)	0.01
≥5	2948	153 (5.19)	1.14	0.77 (0.63–0.94)	0.01	0.72 (0.59–0.88)	<0.001
≥6	2650	138 (5.21)	1.14	0.77 (0.63–0.95)	0.01	0.71 (0.58–0.87)	<0.001
≥7	2392	125 (5.23)	1.14	0.77 (0.63–0.96)	0.02	0.70 (0.57–0.87)	<0.001
≥8	2179	109 (5.00)	1.09	0.74 (0.59–0.92)	0.01	0.66 (0.53–0.82)	<0.001
≥9	1990	99 (4.97)	1.09	0.74 (0.59–0.93)	0.01	0.65 (0.52–0.82)	<0.001
≥10	1826	89 (4.87)	1.07	0.72 (0.57–0.92)	0.01	0.64 (0.50–0.81)	<0.001
≥11	1673	79 (4.72)	1.03	0.70 (0.54–0.90)	0.01	0.62 (0.48–0.79)	<0.001
≥12	1531	69 (4.51)	0.98	0.67 (0.51–0.87)	<0.001	0.58 (0.45–0.76)	<0.001
≥13	1424	61 (4.28)	0.93	0.63 (0.48–0.83)	<0.001	0.55 (0.41–0.72)	<0.001
≥14	1321	52 (3.94)	0.85	0.58 (0.43–0.77)	<0.001	0.50 (0.37–0.67)	<0.001
≥15	1227	46 (3.75)	0.81	0.55 (0.40–0.75)	<0.001	0.47 (0.35–0.65)	<0.001
≥16	1147	43 (3.75)	0.81	0.55 (0.40–0.75)	<0.001	0.47 (0.34–0.64)	<0.001
≥17	1086	40 (3.68)	0.79	0.54 (0.39–0.75)	<0.001	0.46 (0.33–0.64)	<0.001
≥18	1024	39 (3.81)	0.82	0.56 (0.40–0.78)	<0.001	0.47 (0.34–0.66)	<0.001
≥19	960	37 (3.85)	0.83	0.56 (0.40–0.79)	<0.001	0.47 (0.33–0.67)	<0.001
≥20	913	35 (3.83)	0.83	0.56 (0.40–0.80)	<0.001	0.47 (0.33–0.67)	<0.001
≥21	872	35 (4.01)	0.87	0.59 (0.41–0.83)	<0.001	0.49 (0.35–0.70)	<0.001
≥22	830	33 (3.98)	0.86	0.58 (0.41–0.84)	<0.001	0.49 (0.34–0.71)	<0.001
≥23	798	31 (3.88)	0.84	0.57 (0.39–0.82)	<0.001	0.48 (0.33–0.69)	<0.001
≥24	751	30 (3.99)	0.86	0.59 (0.40–0.85)	0.01	0.48 (0.33–0.70)	<0.001
≥25	714	29 (4.06)	0.88	0.60 (0.41–0.87)	0.01	0.49 (0.33–0.72)	<0.001
≥26	674	29 (4.30)	0.93	0.63 (0.43–0.93)	0.02	0.52 (0.35–0.76)	<0.001
≥27	642	28 (4.36)	0.95	0.64 (0.44–0.95)	0.03	0.53 (0.36–0.78)	<0.001
≥28	613	27 (4.40)	0.95	0.65 (0.44–0.96)	0.03	0.52 (0.35–0.78)	<0.001
≥29	595	25 (4.20)	0.91	0.62 (0.41–0.93)	0.02	0.50 (0.33–0.76)	<0.001
≥30	572	23 (4.02)	0.87	0.59 (0.39–0.91)	0.02	0.47 (0.31–0.73)	<0.001

AT group, patients with acupuncture exposure ≥2 times within the first year post-diagnosis; HR, hazard ratios; IR, incidence rate; Non-AT group, patients not exposed to acupuncture within the first year post-diagnosis; PS, propensity score; PY, person-year.

*Cox proportional hazards models were used to estimate hazard ratios, adjusted for age, sex, residential area, income level, disability grade, CHA_2DS_2-VASc score, Charlson Comorbidity Index, and comorbidities (hypertension, diabetes mellitus, dyslipidemia, myocardial infarction, peripheral artery disease, chronic obstructive pulmonary disease, and cancer).

Supplementary Table S3. Hazard ratios based on the number of exposures to acupuncture for all-cause mortality.

	Total, N	Event, n (%)	IR (per 100PY)	PS-matched HR (95% CI)	P	Adjusted HR (95% CI)*	P
Non-AT group	4315	900 (20.86)	4.7	1 [Ref]		1 [Ref]	
AT group							
≥2	4315	695 (16.11)	3.53	0.75 (0.68–0.83)	<0.001	0.73 (0.66–0.81)	<0.001
≥3	3761	616 (16.38)	3.59	0.76 (0.69–0.85)	<0.001	0.73 (0.66–0.81)	<0.001
≥4	3290	541 (16.44)	3.61	0.77 (0.69–0.85)	<0.001	0.73 (0.65–0.81)	<0.001
≥5	2948	485 (16.45)	3.61	0.77 (0.69–0.86)	<0.001	0.71 (0.64–0.79)	<0.001
≥6	2650	426 (16.08)	3.52	0.75 (0.67–0.84)	<0.001	0.69 (0.61–0.77)	<0.001
≥7	2392	387 (16.18)	3.54	0.75 (0.67–0.85)	<0.001	0.68 (0.60–0.77)	<0.001
≥8	2179	351 (16.11)	3.52	0.75 (0.66–0.85)	<0.001	0.67 (0.59–0.76)	<0.001
≥9	1990	325 (16.33)	3.57	0.76 (0.67–0.86)	<0.001	0.67 (0.59–0.76)	<0.001
≥10	1826	296 (16.21)	3.54	0.75 (0.66–0.86)	<0.001	0.67 (0.58–0.76)	<0.001
≥11	1673	268 (16.02)	3.5	0.74 (0.65–0.85)	<0.001	0.66 (0.57–0.75)	<0.001
≥12	1531	240 (15.68)	3.41	0.73 (0.63–0.84)	<0.001	0.64 (0.55–0.74)	<0.001
≥13	1424	214 (15.03)	3.25	0.69 (0.60–0.80)	<0.001	0.61 (0.52–0.70)	<0.001
≥14	1321	192 (14.53)	3.14	0.67 (0.57–0.78)	<0.001	0.59 (0.50–0.68)	<0.001
≥15	1227	179 (14.59)	3.14	0.67 (0.57–0.79)	<0.001	0.58 (0.50–0.68)	<0.001
≥16	1147	166 (14.47)	3.11	0.66 (0.56–0.78)	<0.001	0.57 (0.48–0.67)	<0.001
≥17	1086	157 (14.46)	3.11	0.66 (0.56–0.78)	<0.001	0.57 (0.48–0.67)	<0.001
≥18	1024	149 (14.55)	3.13	0.67 (0.56–0.79)	<0.001	0.57 (0.48–0.67)	<0.001
≥19	960	142 (14.79)	3.19	0.68 (0.57–0.81)	<0.001	0.57 (0.48–0.68)	<0.001
≥20	913	134 (14.68)	3.17	0.67 (0.56–0.81)	<0.001	0.57 (0.48–0.68)	<0.001
≥21	872	128 (14.68)	3.17	0.67 (0.56–0.81)	<0.001	0.57 (0.47–0.68)	<0.001
≥22	830	123 (14.82)	3.2	0.68 (0.57–0.82)	<0.001	0.58 (0.48–0.70)	<0.001
≥23	798	118 (14.79)	3.19	0.68 (0.56–0.82)	<0.001	0.57 (0.47–0.69)	<0.001
≥24	751	112 (14.91)	3.22	0.69 (0.56–0.83)	<0.001	0.57 (0.47–0.69)	<0.001
≥25	714	108 (15.13)	3.28	0.70 (0.57–0.85)	<0.001	0.57 (0.47–0.70)	<0.001
≥26	674	103 (15.28)	3.31	0.71 (0.58–0.86)	<0.001	0.58 (0.47–0.71)	<0.001
≥27	642	101 (15.73)	3.42	0.73 (0.59–0.89)	<0.001	0.59 (0.48–0.73)	<0.001
≥28	613	96 (15.66)	3.4	0.72 (0.59–0.89)	<0.001	0.58 (0.47–0.72)	<0.001
≥29	595	92 (15.46)	3.35	0.71 (0.58–0.88)	<0.001	0.58 (0.47–0.72)	<0.001
≥30	572	90 (15.73)	3.41	0.73 (0.59–0.90)	<0.001	0.58 (0.47–0.72)	<0.001

AT group, patients with acupuncture exposure ≥2 times within the first year post-diagnosis; HR, hazard ratios; IR, incidence rate; Non-AT group, patients not exposed to acupuncture within the first year post-diagnosis; PS, propensity score; PY, person-year.

*Cox proportional hazards models were used to estimate hazard ratios, adjusted for age, sex, residential area, income level, disability grade, CHA_2DS_2-VASc score, Charlson Comorbidity Index, and comorbidities (hypertension, diabetes mellitus, dyslipidemia, myocardial infarction, peripheral artery disease, chronic obstructive pulmonary disease, and cancer).

Supplementary Table S4. Patient demographics and clinical characteristics in sensitivity analysis

	Before PS matching			After PS matching		
	Non-AT group (n = 4,028)	AT group (n = 5,789)	SMD	Non-AT group (n = 1,857)	AT group (n = 1,857)	SMD
**Age, mean (SD)**	66.77 (14.66)	67.54 (11.86)	0.057	66.41 (15.09)	66.50 (13.21)	0.006
**Age, n (%)**			0.211			0.017
<65	1627 (40.4)	2035 (35.2)		777 (41.8)	772 (41.6)	
<75	988 (24.5)	1974 (34.1)		430 (23.2)	443 (23.9)	
≥75	1413 (35.1)	1780 (30.7)		650 (35.0)	642 (34.6)	
**Sex, n (%)**			0.417			0.012
Male	2213 (54.9)	2005 (34.6)		832 (44.8)	821 (44.2)	
Female	1815 (45.1)	3784 (65.4)		1025 (55.2)	1036 (55.8)	
**Residential area, n (%)**			0.152			0.017
Metropolitan	1711 (42.5)	2032 (35.1)		767 (41.3)	780 (42.0)	
Urban	716 (17.8)	1185 (20.5)		293 (15.8)	295 (15.9)	
Rural	1597 (39.7)	2567 (44.4)		797 (42.9)	782 (42.1)	
**Income, n (%)**			0.055			0.005
1	779 (22.3)	1110 (21.7)		345 (18.6)	344 (18.5)	
2	833 (23.8)	1121 (21.9)		389 (20.9)	386 (20.8)	
3	1885 (53.9)	2891 (56.4)		1123 (60.5)	1127 (60.7)	
**Disability grade, n (%)**			0.148			0.006
Non-disabled	3173 (78.8)	4814 (83.2)		1682 (90.6)	1679 (90.4)	
Mild	475 (11.8)	648 (11.2)		111 (6.0)	112 (6.0)	
Severe	380 (9.4)	327 (5.6)		64 (3.4)	66 (3.6)	
**CHA_2DS_2-VAS_C score, mean (SD)**	3.01 (1.73)	3.32 (1.66)	0.184	3.05 (1.77)	3.00 (1.71)	0.030
**CHA_2DS_2-VAS_C score, n (%)**			0.202			0.011
0	227 (5.6)	177 (3.1)		109 (5.9)	112 (6.0)	
1	621 (15.4)	671 (11.6)		299 (16.1)	301 (16.2)	
2	820 (20.4)	1051 (18.2)		339 (18.3)	343 (18.5)	
≥3	2360 (58.6)	3890 (67.2)		1110 (59.8)	1101 (59.3)	
**CCI, mean (SD)**	2.58 (2.45)	2.80 (2.19)	0.096	2.32 (2.24)	2.27 (2.11)	0.022
**CCI, n (%)**			0.216			0.023
0	786 (19.5)	696 (12.0)		426 (22.9)	433 (23.3)	
1	854 (21.2)	1196 (20.7)		411 (22.1)	416 (22.4)	
2	704 (17.5)	1116 (19.3)		290 (15.6)	298 (16.0)	
≥3	1684 (41.8)	2781 (48.0)		730 (39.3)	710 (38.2)	
**Comorbidities, n (%)**						
Hypertension	2838 (70.5)	4292 (74.1)	0.082	1384 (74.5)	1374 (74.0)	0.012
Diabetes mellitus	1388 (34.5)	1901 (32.8)	0.034	596 (32.1)	584 (31.4)	0.014
Dyslipidemia	1615 (40.1)	2600 (44.9)	0.098	735 (39.6)	738 (39.7)	0.003
MI	280 (7.0)	308 (5.3)	0.068	72 (3.9)	71 (3.8)	0.003
PAD	562 (14.0)	1170 (20.2)	0.167	232 (12.5)	231 (12.4)	0.002
COPD	291 (7.2)	364 (6.3)	0.037	61 (3.3)	54 (2.9)	0.022
Cancer	308 (7.6)	357 (6.2)	0.058	61 (3.3)	60 (3.2)	0.003

AT group, patients with acupuncture exposure ≥2 times within the first year post-diagnosis; CCI, Charlson comorbidity index; COPD, chronic obstructive pulmonary disease; MI, myocardial infarction; Non-AT group, patients who have never been exposed to acupuncture after diagnosis; PAD, peripheral artery disease; PS, propensity score; SD, standard deviation; SMD, standardized mean difference.

Supplementary Table S5. Hazard ratios of heart failure patients in sensitivity analysis

Mortality	Event, n (%)	IR (per 100PY)	PS-matched HR (95% CI)	P	Adjusted HR (95% CI)*	P
Circulatory system disease						
Non-AT group	177 (9.53)	2.36	1 [Ref]		1 [Ref]	
AT group	104 (5.60)	1.22	0.53 (0.41–0.67)	<0.001	0.47 (0.37–0.60)	<0.001
All-cause						
Non-AT group	573 (30.86)	7.63	1 [Ref]		1 [Ref]	
AT group	303 (16.32)	3.56	0.47 (0.41–0.54)	<0.001	0.43 (0.37–0.49)	<0.001

AT group, patients with acupuncture exposure ≥2 times within the first year post-diagnosis; HR, hazard ratios; IR, incidence rate; Non-AT group, patients who have never been exposed to acupuncture after diagnosis; PS, propensity score; PY, person-year.

*Cox proportional hazards models were used to estimate hazard ratios, adjusted for age, sex, residential area, income level, disability grade, CHA_2DS_2-VASc score, Charlson Comorbidity Index, and comorbidities (hypertension, diabetes mellitus, dyslipidemia, myocardial infarction, peripheral artery disease, chronic obstructive pulmonary disease, and cancer).

Supplementary Table S6. Hazard ratios stratified by quartiles of acupuncture treatment exposure in the sensitivity analysis

Mortality	Total, N	Event, n (%)	IR (per 100PY)	PS-matched HR (95% CI)	P	Adjusted HR (95% CI)*	P
Circulatory system disease							
Non-AT group	1857	177 (9.53)	2.36	1 [Ref]		1 [Ref]	
AT group							
AT-1	603	31 (5.14)	1.11	0.48 (0.33–0.70)	<0.001	0.51 (0.35–0.74)	<0.001
AT-2	403	25 (6.20)	1.37	0.59 (0.39–0.89)	0.01	0.56 (0.36–0.85)	0.01
AT-3	417	30 (7.19)	1.6	0.69 (0.47–1.01)	0.06	0.57 (0.39–0.85)	0.01
AT-4	434	18 (4.15)	0.89	0.38 (0.24–0.62)	<0.001	0.29 (0.18–0.47)	<0.001
All-cause							
Non-AT group	1857	573 (30.86)	7.63	1 [Ref]		1 [Ref]	
AT group							
AT-1	603	85 (14.10)	3.05	0.41 (0.32–0.51)	<0.001	0.43 (0.34–0.54)	<0.001
AT-2	403	69 (17.12)	3.77	0.50 (0.39–0.64)	<0.001	0.48 (0.37–0.62)	<0.001
AT-3	417	81 (19.42)	4.33	0.57 (0.45–0.72)	<0.001	0.49 (0.39–0.62)	<0.001
AT-4	434	68 (15.67)	3.37	0.45 (0.35–0.57)	<0.001	0.34 (0.27–0.44)	<0.001

AT group, patients with acupuncture exposure ≥2 times within the first year post-diagnosis; AT-1, <Q1; AT-2, ≥Q1 and <Q2; AT-3, ≥Q2 and <Q3; AT-4, ≥Q3; HR, hazard ratios; IR, incidence rate; Non-AT group, patients who have never been exposed to acupuncture after diagnosis; PS, propensity score; PY, person-year.

*Cox proportional hazards models were used to estimate hazard ratios, adjusted for age, sex, residential area, income level, disability grade, CHA_2DS_2-VASc score, Charlson Comorbidity Index, and comorbidities (hypertension, diabetes mellitus, dyslipidemia, myocardial infarction, peripheral artery disease, chronic obstructive pulmonary disease, and cancer).

Supplementary Table S7. Hazard ratios based on the number of exposures to acupuncture treatment for circulatory system disease-related mortality in the sensitivity analysis

	Total, N	Event, n (%)	IR (per 100PY)	PS-matched HR (95% CI)	P	Adjusted HR (95% CI)*	P
Non-AT group	1857	177 (9.53%)	2.36	1 [Ref]		1 [Ref]	
AT group							
≥2	1857	104 (5.60%)	1.22	0.53(0.41–0.67)	<0.001	0.47 (0.37–0.60)	<0.001
≥3	1611	92 (5.71%)	1.25	0.54(0.42–0.69)	<0.001	0.47 (0.37–0.61)	<0.001
≥4	1405	83 (5.91%)	1.3	0.56(0.43–0.72)	<0.001	0.48 (0.37–0.63)	<0.001
≥5	1254	73 (5.82%)	1.28	0.55(0.42–0.72)	<0.001	0.46 (0.35–0.61)	<0.001
≥6	1140	67 (5.88%)	1.29	0.55(0.42–0.73)	<0.001	0.46 (0.35–0.61)	<0.001
≥7	1019	60 (5.89%)	1.29	0.56(0.42–0.74)	<0.001	0.45 (0.34–0.61)	<0.001
≥8	933	55 (5.89%)	1.29	0.55(0.41–0.75)	<0.001	0.44 (0.33–0.61)	<0.001
≥9	851	48 (5.64%)	1.23	0.53(0.39–0.73)	<0.001	0.42 (0.30–0.58)	<0.001
≥10	776	42 (5.41%)	1.18	0.51(0.36–0.71)	<0.001	0.40 (0.28–0.56)	<0.001
≥11	722	39 (5.40%)	1.18	0.51(0.36–0.72)	<0.001	0.40 (0.28–0.57)	<0.001
≥12	663	35 (5.28%)	1.15	0.50(0.35–0.71)	<0.001	0.39 (0.27–0.56)	<0.001
≥13	613	30 (4.89%)	1.06	0.46(0.31–0.67)	<0.001	0.35 (0.24–0.52)	<0.001
≥14	567	23 (4.06%)	0.87	0.38(0.24–0.58)	<0.001	0.29 (0.19–0.45)	<0.001
≥15	529	21 (3.97%)	0.85	0.37(0.23–0.58)	<0.001	0.28 (0.18–0.44)	<0.001
≥16	498	20 (4.02%)	0.86	0.37(0.24–0.59)	<0.001	0.28 (0.18–0.44)	<0.001
≥17	463	19 (4.10%)	0.88	0.38(0.24–0.61)	<0.001	0.29 (0.18–0.46)	<0.001
≥18	434	18 (4.15%)	0.89	0.39(0.24–0.62)	<0.001	0.28 (0.18–0.46)	<0.001
≥19	404	16 (3.96%)	0.85	0.37(0.22–0.61)	<0.001	0.27 (0.16–0.45)	<0.001
≥20	385	15 (3.90%)	0.84	0.36(0.21–0.61)	<0.001	0.27 (0.16–0.46)	<0.001
≥21	368	15 (4.08%)	0.88	0.38(0.22–0.64)	<0.001	0.28 (0.17–0.48)	<0.001
≥22	351	14 (3.99%)	0.86	0.37(0.22–0.64)	<0.001	0.28 (0.16–0.48)	<0.001
≥23	342	13 (3.80%)	0.82	0.35(0.20–0.62)	<0.001	0.26 (0.15–0.46)	<0.001
≥24	327	13 (3.98%)	0.85	0.37(0.21–0.65)	<0.001	0.27 (0.15–0.48)	<0.001
≥25	312	12 (3.85%)	0.83	0.36(0.20–0.64)	0.001	0.27 (0.15–0.48)	<0.001
≥26	291	12 (4.12%)	0.89	0.39(0.22–0.69)	0.001	0.29 (0.16–0.52)	<0.001
≥27	280	12 (4.29%)	0.93	0.40(0.22–0.72)	0.001	0.30 (0.17–0.54)	<0.001
≥28	268	12 (4.48%)	0.97	0.42(0.24–0.75)	0.001	0.31 (0.17–0.55)	<0.001
≥29	260	11 (4.23%)	0.92	0.40(0.22–0.73)	0.001	0.29 (0.16–0.54)	<0.001
≥30	252	10 (3.97%)	0.86	0.37(0.20–0.70)	0.001	0.27 (0.14–0.51)	<0.001

AT group, patients with acupuncture exposure ≥2 times within the first year post-diagnosis; HR, hazard ratios; IR, incidence rate; Non-AT group, patients who have never been exposed to acupuncture after diagnosis; PS, propensity score; PY, person-year.

*Cox proportional hazards models were used to estimate hazard ratios, adjusted for age, sex, residential area, income level, disability grade, CHA_2DS_2-VASc score, Charlson Comorbidity Index, and comorbidities (hypertension, diabetes mellitus, dyslipidemia, myocardial infarction, peripheral artery disease, chronic obstructive pulmonary disease, and cancer).

Supplementary Table S8. Hazard ratios based on the number of exposures to acupuncture treatment for all-cause mortality in sensitivity analysis

	Total, N	Event, n (%)	IR (per 100PY)	PS-matched HR (95% CI)	P	Adjusted HR (95% CI)*	P
Non-AT group	1857	573 (30.86)	7.63	1 [Ref]		1 [Ref]	
AT group							
≥2	1857	303 (16.32)	3.56	0.47 (0.41–0.54)	<0.001	0.43 (0.37–0.49)	<0.001
≥3	1611	272 (16.88)	3.7	0.49 (0.43–0.57)	<0.001	0.44 (0.38–0.51)	<0.001
≥4	1405	243 (17.30)	3.79	0.50 (0.43–0.58)	<0.001	0.44 (0.38–0.51)	<0.001
≥5	1254	218 (17.38)	3.81	0.51 (0.43–0.59)	<0.001	0.43 (0.37–0.50)	<0.001
≥6	1140	195 (17.11)	3.75	0.50 (0.42–0.58)	<0.001	0.42 (0.36–0.50)	<0.001
≥7	1019	175 (17.17)	3.77	0.50 (0.42–0.59)	<0.001	0.41 (0.35–0.49)	<0.001
≥8	933	162 (17.36)	3.8	0.50 (0.42–0.60)	<0.001	0.41 (0.34–0.49)	<0.001
≥9	851	149 (17.51)	3.83	0.51 (0.43–0.61)	<0.001	0.41 (0.34–0.49)	<0.001
≥10	776	134 (17.27)	3.78	0.50 (0.42–0.60)	<0.001	0.40 (0.33–0.48)	<0.001
≥11	722	124 (17.17)	3.75	0.50 (0.41–0.60)	<0.001	0.40 (0.33–0.48)	<0.001
≥12	663	112 (16.89)	3.69	0.49 (0.40–0.60)	<0.001	0.39 (0.32–0.48)	<0.001
≥13	613	97 (15.82)	3.42	0.46 (0.37–0.56)	<0.001	0.36 (0.29–0.44)	<0.001
≥14	567	81 (14.29)	3.06	0.41 (0.32–0.51)	<0.001	0.32 (0.25–0.40)	<0.001
≥15	529	77 (14.56)	3.12	0.42 (0.33–0.53)	<0.001	0.32 (0.26–0.41)	<0.001
≥16	498	74 (14.86)	3.19	0.42 (0.34–0.54)	<0.001	0.33 (0.26–0.41)	<0.001
≥17	463	70 (15.12)	3.24	0.43 (0.34–0.55)	<0.001	0.33 (0.26–0.42)	<0.001
≥18	434	68 (15.67)	3.37	0.45 (0.35–0.57)	<0.001	0.34 (0.27–0.43)	<0.001
≥19	404	63 (15.59)	3.35	0.45 (0.35–0.58)	<0.001	0.34 (0.26–0.43)	<0.001
≥20	385	58 (15.06)	3.24	0.43 (0.33–0.56)	<0.001	0.33 (0.25–0.43)	<0.001
≥21	368	56 (15.22)	3.27	0.44 (0.33–0.57)	<0.001	0.33 (0.25–0.44)	<0.001
≥22	351	53 (15.10)	3.25	0.43 (0.33–0.57)	<0.001	0.33 (0.25–0.44)	<0.001
≥23	342	52 (15.20)	3.27	0.44 (0.33–0.58)	<0.001	0.33 (0.25–0.44)	<0.001
≥24	327	50 (15.29)	3.29	0.44 (0.33–0.58)	<0.001	0.33 (0.25–0.44)	<0.001
≥25	312	47 (15.06)	3.24	0.43 (0.32–0.58)	<0.001	0.33 (0.24–0.44)	<0.001
≥26	291	46 (15.81)	3.42	0.46 (0.34–0.61)	<0.001	0.35 (0.26–0.46)	<0.001
≥27	280	46 (16.43)	3.56	0.48 (0.35–0.64)	<0.001	0.36 (0.27–0.48)	<0.001
≥28	268	46 (17.16)	3.74	0.50 (0.37–0.67)	<0.001	0.37 (0.28–0.50)	<0.001
≥29	260	43 (16.54)	3.58	0.48 (0.35–0.65)	<0.001	0.36 (0.27–0.49)	<0.001
≥30	252	42 (16.67)	3.61	0.48 (0.35–0.66)	<0.001	0.36 (0.27–0.49)	<0.001

AT group, patients with acupuncture exposure ≥2 times within the first year post-diagnosis; HR, hazard ratios; IR, incidence rate; Non-AT group, patients who have never been exposed to acupuncture after diagnosis; PS, propensity score; PY, person-year.

*Cox proportional hazards models were used to estimate hazard ratios, adjusted for age, sex, residential area, income level, disability grade, CHA_2DS_2-VASc score, Charlson Comorbidity Index, and comorbidities (hypertension, diabetes mellitus, dyslipidemia, myocardial infarction, peripheral artery disease, chronic obstructive pulmonary disease, and cancer).
